# Quality by Design (QbD) and Design of Experiments (DOE) as a Strategy for Tuning Lipid Nanoparticle Formulations for RNA Delivery

**DOI:** 10.3390/biomedicines11102752

**Published:** 2023-10-11

**Authors:** Lidia Gurba-Bryśkiewicz, Wioleta Maruszak, Damian A. Smuga, Krzysztof Dubiel, Maciej Wieczorek

**Affiliations:** Medicinal Chemistry Department, Celon Pharma S.A., Marymoncka 15, 05-152 Kazuń Nowy, Poland; wioleta.maruszak@celonpharma.com (W.M.); damian.smuga@celonpharma.com (D.A.S.); krzysztof.dubiel@celonpharma.com (K.D.); maciej.wieczorek@celonpharma.com (M.W.)

**Keywords:** quality by design (QbD), design of experiment (DOE), lipid nanoparticle (LNP), RNA delivery

## Abstract

The successful development of nonviral delivery systems for nucleic acids has been reported extensively over the past years. Increasingly employed to improve the delivery efficiency and therapeutic efficacy of RNA are lipid nanoparticles (LNPs). Many of the various critical formulation parameters can affect the quality attributes and effectiveness of these nano-formulations. Therefore, the systematic drug development approach (QbD) and multivariate design and statistical analysis (DOE) can be very helpful and recommended for the optimization of the composition and production of RNA–LNPs. This review addresses the concepts and applications of QbD and/or DOE for the development of lipid nanoparticles for the delivery of different types of RNA, reporting examples published in the ten recent years presenting the latest trends and regulatory requirements as well as the modern mathematical and statistical design methods. As the topic explored in this review is a novel approach, the full QbD has been described in only a few papers, and a few refer only to some aspects of QbD. In contrast, the DOE approach has been used in most of the optimization works. Different approaches and innovations in DOE have been observed. Traditional statistical tests and modeling (ANOVA, regression analysis) are slowly being replaced by artificial intelligence and machine learning methods.

## 1. Introduction

In recent years, important developments in the pharmaceutical industry have occurred. Modern systems have been developed to ensure product quality in manufacturing information, quality management systems, and risk management [1,2,3]. These tools allow drug manufacturers to detect, analyze, correct, and prevent problems while continuously improving the drug manufacturing process [4,5,6]. Supporting this modernization in the rules used to manage drug manufacturing and product quality, in 2002, the FDA introduced the regulatory environment of the Current Good Manufacturing Practices (cGMP) [7,8].

After the publication of the International Conference on Harmonization of Technical Requirements for Registration of Pharmaceuticals for Human Use considerations (ICH) guidelines Q8 (R2), Q9, Q10, and Q11 [9,10,11,12], regulatory agencies (Food and Drug Administration—FDA and European Medicines Agency—EMA) approved the Quality by Design (QbD) approach. The ICH Q8 guideline recommended the integration of the “quality by design (QbD)” concept into the pharmaceutical industry [9].

The development of safe and efficient new drugs is a long, difficult, and expensive process. Using the new QbD approach, which ensures that the quality is provided by manufacturing instead of by-product testing, decreases the costs of product optimization. QbD studies may provide some income to the drug manufacturers and benefits for patient safety—ensuring that they will obtain higher-quality drugs in a shorter time.

As defined by ICH Q8 [9], QbD is a systematic drug development approach based on predefined goals, an understanding of the product and processes, and sound science and quality risk management.

In recent decades, research related to nanosystems has started a technological revolution in medicines, especially in RNA delivery. Some researchers apply QbD in the development of drug RNA-LNP systems [13,14,15,16,17,18,19,20,21,22,23,24,25,26,27,28,29].

This review summarized the QbD concepts and applications to the development of pharmaceutical products containing RNA-LNPs. The second section describes the concepts of QbD, pharmaceutical QbD implementation, risk assessment, design of experiment (DoE), and process analytical technology (PAT). Afterward, the use of the QbD and DOE approach in the development of lipid nanoparticles is shown through examples of works published in the last 10 years.

## 2. Quality by Design Concept

**Figure 1:**
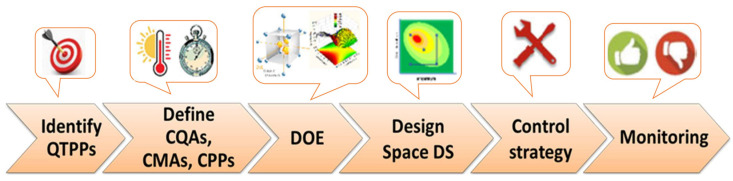
Scheme of quality by design for a development pharmaceutical product.

Generally, the steps for implementing QbD in the development of pharmaceutical products are similar and include (Figure 1) the use of risk assessment to identify risk parameters, defining the main quality target product profile (QTTP), identifying parameters that influence the process performance, carrying out a DOE and definition of dependency of critical quality attributes (CQAs) to critical material attributes (CMAs) and critical process parameters (CPPs), defining a process design space that originates a final product with the desired QTPP, developing a risk control strategy to identify the causes of variability, and continuous monitoring and improving the manufacturing process [2,3,4,8,9,30].

### 2.1. Quality Target Product Profile (QTTP)

A quality target product profile (QTPP) consists of quantitative support for clinical safety and efficiency, and it makes a foundation to design and optimize a formulation or manufacturing process. The QTPP is a set of the attributes and characteristics of a product that determine its quality. According to the FDA, the QTPP is more focused on the chemical, manufacturing, and control stages of development [7,9].

The QTPP defines the quantitative characteristic of the drug and refers to specifications such as the dosage form, application way, packing, appearance, and diagnosis. Examples of QTPP are clinical use, administration route, therapeutic dosage, pharmaceutical dosage form, drug delivery system, packing container, factors affecting pharmacokinetic parameters, and quality criteria of the final product, such as stability during storage, sterility, and drug release. The QTPP only includes the product properties related to the patient. QTPP served as a guide for the optimization of formulation and process parameters to ensure that CQAs were within the desired range [1,2,3,4,8,30].

### 2.2. Critical Quality Attributes (CQA)

CQAs are physical, chemical, biological, or microbiological attributes/characteristics that should be controlled to ensure product quality [9]. CQAs are parameters that affect QTPP and are critical to product quality. These are generally related to the selection of correct amounts of excipients and drugs [1,2,3,4,8,30].

### 2.3. Critical Process Parameters (CPP)

CPPs are production parameters that affect CQAs and should be controlled. For example, parameters that interfere with the quality of the final product should be monitored during the production process. A parameter represents a measured or calculated characteristic of a system or process. These are parameters of the manufacturing system and are usually properties of materials or processes that affect production, such as temperature and composition.

### 2.4. Critical Material Attributes (CMAs)

CMAs are properties of materials that must reach adequate limits to guarantee the quality of excipients, drugs, and other materials used during the process.

It is important to understand the difference between CQAs (output data) and CMAs (input data) during development. For example, CQAs of an intermediate may become CMAs of an intermediate in the next production step. Moreover, based on the QbD approach, CMAs and CPPs may differ in the defined design space without significantly interfering with CQAs. Additionally, following the ICH guideline “Quality Risk Management” (ICH Q9), the identification of possible causes of process variability and effective risk analysis is of key importance during process optimization. This procedure can be performed in the initial or final steps, repeating or redefining it as needed. Thus, the quality of the final product is verified by the previous experience in the determination of CQAs, CPPs, and risk assessment [8,10,31].

### 2.5. Risk Assessment

Risk assessment is used to identify what can go wrong, the likelihood of it going wrong, and the consequences. According to ICH Q9, risk control is related to procedures that should be adopted to reduce risks [10]. This guidance provides examples of commonly used risk management tools. At this stage, it is necessary to decide which risks can be accepted and which should be minimized. Analysis of variance (ANOVA) or multiple regression analysis is used for risk assessment based on experimental data. A mathematical equation for the relationship between variables can be derived using multiple regression analysis. While using ANOVA, the statistical significance of the influence of each factor and interaction effects is assessed [31]. In the final stage, the results should be monitored using a risk assessment, taking into account previous knowledge and experience. Identifying QTPP, CQA, and CPP requires prior experience and knowledge as various risk assessment tools are used, including risk filtering, Ishikawa diagram, and failure mode and effects analysis [4,10].

### 2.6. Design Space (DS)

The design space (DS) has an important place in the pharmaceutical industry. The design space defines the multivariate functional relationships between CQA and CPP and includes their relationships. These relationships can be found by applying risk assessment, design of experiments (DOE), and modeling.

The design space is specific to a unit operation or a single manufacturing process and defines operating process parameters (such as moisture ratio) that are known to affect product quality. DS can also be considered as a link between CQA and CPP [32]. It is a way to show the development of understanding of a process, and the benefits of creating a design space are obvious. However, one of the challenges for using DS effectively is the cost of creating it. The most important point in developing the design space is to demonstrate or determine that the unclassified parameters excluded from the DOE are non-critical process parameters and, therefore, do not interact. Therefore, DOE screening can be used to identify interactions between process parameters. In the absence of interaction, single-variable intervals can be directly added to the design space as non-critical parameters.

### 2.7. Control Strategy

A control strategy is a very important step to secure the process performance and product quality. It should be carefully planned based on product and process information [11]. In the QbD approach, a control strategy is performed during product development and allows a deeper understanding of the product and process. Control strategy options with QbD are easier for areas requiring more time and specialty knowledge by delivering more information than the standard approach. In this novelty approach to pharmaceutical quality, the finished product quality is built by identifying and controlling an optimal range of formulation and manufacturing variables. Finished products are finally tested to confirm the product quality [33].

The development of an effective control strategy should be risk-based to ensure that product quality requirements are met. The development of the control strategy starts with the QTPP. The first studies are focused on the characterization of the active ingredient and important physical, chemical, biological, and microbiological properties of the formulation. The development of the process is also defined in this phase. For example, if the active ingredient has low solubility in water, the immediate-release tablet form must ensure sufficient dissolution of the drug. Toxicity studies performed during the early stages provide a baseline assessment of the impurity profile of the active ingredient. Understanding impurity generation and removal, at which stages elements of the control strategy will take place, and the development of acceptance criteria and methods to be included in the specifications will provide an important point of view. Although a control strategy should be developed using these steps, it can also be developed using the quality risk management principles defined in ICH Q9 [10,33].

During production, the continuous control strategy enables understanding and stabilization of the process. It is very important to modify the pharmaceutical quality system for supporting to the use of a QbD approach in the control strategy. In the commercial manufacturing process, it should be made possible to update, modify, or continuously improve the control strategy. When product information, documents, and operating procedures must be changed, the pharmaceutical quality system should provide the appropriate technical and administrative audits required to obtain the necessary reviews and approvals [33].

## 3. QbD and DOE in the Development of the RNA-LNP System

**Figure 2:**
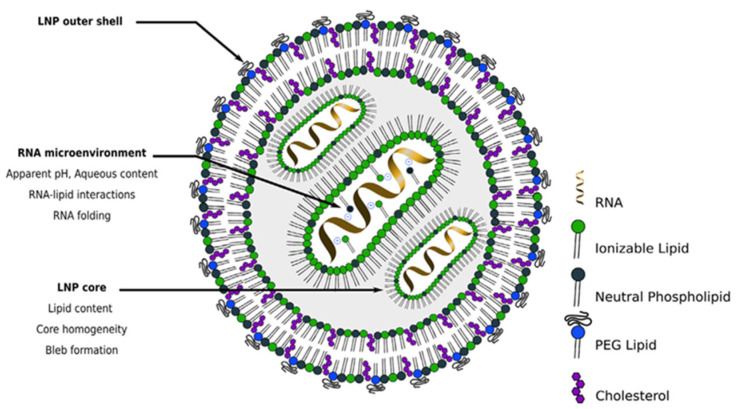
Scheme of the hypothetical structure of an RNA-LNP system (adapted from [13]).

QbD implementation in the development of pharmaceutical products containing RNA-LNPs is closely connected to the structure (Figure 2) and the main therapeutic goal of the nanosystem.

The definition of the main steps of the QbD approach is similar to different published research (see Table 1) and includes generally the following parameters [14]:

- QTPP: safety and efficacy.
- CQAs: z-average, PDI, zeta potential, encapsulation efficiency, loading efficiency, stability, shelf life, storage conditions, and lipids contents.
- CMAs: N/P ratio, lipids type, non-toxicity, biodegradability.
- Critical process parameters (CPPs): temperature, microfluidics, filtration.

In the DOE, a wide range of different statistical approaches and modeling methods were used.

The following table shows examples of recent studies using the QbD and/or DOE approach to optimize formulations containing RNA-LNPs (Table 1). Particular information about optimized variables and their tested range applied in the DOE approach was described in Section DOE Approach—Design Models, Variables, and Range. More detailed results of these papers are shown in Supplementary Materials (Table S1).

### DOE Approach—Design Models, Variables, and Range

In the first paper listed in Table 1, Cun J. et al. [34] applied 2^(5−1)^ fractional factorial design (FFD)—five input variables with a center point (each on the three levels): inner water phase and the oil phase volume ratio (0.1; 0.3; 0.5), poly(DL-lactide-glycolide acid) (PLGA) concentration (20.0; 40.0; 60.0 mg/mL), sonication time for the primary emulsification (30.0; 60.0; 90.0 s), siRNA load (35.9; 62.8; 89.7 µg), acetylated bovine serum albumin (Ac-BSA) content in the inner water phase (0.0; 200; 400 mg). Optimized variables were encapsulation efficiency (%EE) of the siRNA, obtained in the range of 2.01% to 51.18%, and particle size in the range of 218.3 nm (standard deviation, SD (*n* = 3) = 0.7 nm) to 257.9 nm (SD = 2.7 nm). Optimal values of significant input parameters are volume ratio 0.46, PLGA concentration 40.7 mg/mL, siRNA load 39 µg, acetylated bovine serum albumin (Ac-BSA) content in the inner water phase 400 µg for low siRNA load (770 ng/mg nanoparticles, SD = 33 ng/mg nanoparticles), volume ratio 0.40, PLGA concentration 42.4 mg/mL, siRNA load 90 µg, acetylated bovine serum albumin (Ac-BSA) content in the inner water phase 400 µg for high siRNA load (2192 ng/mg nanoparticles, SD = 115 ng/mg nanoparticles). The optimal values of %EE were equal to 64.35% (SD = 2.78%) for low siRNA load and 70.63% (SD = 5.75%) for high siRNA load. The optimal average particle size was equal to about 260 nm both for low and high siRNA load. The zeta potentials, additionally measured, were all negative in the range of −45.5 mV to −37.5 mV.

Chen D. et al. [35] optimized siRNA-LNP in terms of the structure of used cationic lipids. The matrix of 70 different compounds of cationic lipids was tested—5 different hydrocarbon chain lengths (C8–C14) and 14 different amine headgroups. Optimized output parameters were: hydrodynamic diameter of particles (obtained in the range of 50 to 130 nm), total siRNA concentration, the percentage of particle-entrapped siRNA and luciferase silencing in vitro, and factor VII silencing in vivo (C57BL/6 mice). The lipids composition of LNPs was 2.0 mg/mL of cationic lipid, 0.28 mg/mL of DSPC, 0.52 mg/mL of cholesterol, and 0.13 mg/mL of mPEG2000-DMG. The concentration of polynucleotide was 0.4 mg/mL in 10 mM citrate buffer, pH 3.0. The dose of LNPs for in vivo factor VII delivery was 1.0 mg/kg (siRNA/body weight), and volume injection was less than 200 μL into the animal. Two main dependencies were identified from the LNPs test: siRNA delivery efficiencies in vitro and in vivo for different amine headgroups are a function of the hydrocarbon chain length, the potency of in vitro did not change significantly with hydrocarbon chain length, the in vivo potency of LNPs decreased with increasing hydrophobicity of the cationic lipids. In addition, based on this optimized approach, seven novel materials with in vivo gene silencing potencies of >90% at a dose of 1.0 mg/kg in mice were discovered.

In turn, Li et al. [36] carried out optimization of lipid-like nanoparticles (LLNs) composed with different derivatives of *N*1,*N*3,*N*5-tris(2-aminoethyl)benzene-1,3,5-tri carboxamide (TT) for mRNA delivery. In the first step, the correlation analysis was performed to choose the most optimal type of TT (seven different compounds were tested). The output variables were: particle size (obtained in the range of 99 nm (SD = 2 nm) to 178 nm (SD = 1 nm), polydispersity index (PDI < 0.2), zeta potential (all nanoparticles were positively charged), entrapment efficiency of mRNA (15–82%), delivery efficiency, and cytotoxicity of TT2-TT8 LLNs-FLuc mRNA in Hep3B cells. At a dose of 1.2 μg/mL of luciferase mRNA, LLNs with TT3 as components characterized statistically significantly higher expression of the firefly luciferase compared to other TT LLNs. A significant positive correlation was observed between transfection efficiency and entrapment efficiency. No significant correlation was noticed between transfection efficiency and particle size, surface charge, and cell viability. In the next steps, the content of other ingredients of TT3-LLNs was optimized. Two separate orthogonal experimental designs, L16(4^4^), were performed for different ranges of tested parameters. Four input variables on four levels were considered. The first design concerned the TT3 mole ratio (30; 40; 50; 60), dioleoyl phosphatidylethanolamine (DOPE) mole ratio (1.25; 2.5; 5.0; 10.0), cholesterol mole ratio (18.5; 28.5; 38.5; 48.5), DMG-PEG2000 mole ratio (0.75; 1.5; 3.0; 6.0), and for the second, the TT3 mole ratio (10; 20; 30; 40), DOPE mole ratio (10.0; 20.0; 30.0; 40.0), cholesterol (Chol) mole ratio (25; 30; 35; 40), DMG-PEG2000 mole ratio (0.0; 0.03; 0.15; 0.75). In both cases, the mRNA delivery efficiency expressed as a relative intensity of luminescence was used as an output variable. In the first optimization, the efficiency was obtained in the range of 20 to 4663, and in the second, obtained in the range of 1917 to 101,863, depending on the composition of TT3-LLNs. The optimal composition of the formulation was TT3/DOPE/Chol/DMG-PEG2000: 20/30/40/0, but the particle size of these LLNs, with a low ratio of DMG-PEG2000, increased dramatically 5 h after. Whereas for formulation TT3/DOPE/Chol/DMG-PEG2000: 20/30/40/0.75 were stable for a minimum of 2 weeks.

In the next paper, Kauffman et al. [37] carried out the optimization of the composition of LNPs Erythropoietin(EPO)-mRNA-loaded with C12-200. Six input variables were considered: C12-200:mRNA weight ratio, phospholipid type and phospholipid, C12-200, cholesterol, and PEG molar contents. The output variables were encapsulation efficiency (%EE), particle size, polydispersity index (PDI), and EPO serum concentration. In the first step, library A was built with a definitive screening design (14 experiments). The most optimal phospholipid type was chosen as a matrix of qualitative variables: tail group (DS, DO) and head group (PC, PE). Quantitative variables were on the three levels: C12-200:mRNA weight ratio (2.5; 5.0; 7.5), C12-200 %mol (40; 50; 60), phospholipid %mol (4; 10; 16), PEG %mol (0.5; 1.5; 2.5), and cholesterol %mol was added to 100% (21.5–55.50). DSPC and DOPE were selected for further optimization. Within library B, Taguchi fractional factorial screening design (18 experiments) was performed with the following input variables: C12-200:mRNA weight ratio (7.5, 10.0, 12.5), C12-200 %mol (30, 35, 40), phospholipid %mol (16, 22, 28), PEG %mol (2.5, 3.0), and cholesterol %mol (from 28.5 to 51.5). The DOPE phospholipid was selected as the most optimal. In the final step (library C, 6 experiments), the C12-200:mRNA weight ratio was optimized in the range of 5.0; 7.5; 10.0; 15.0; 20.0; 25.0. The optimal composition of the formulation was 10:1 C12-200:mRNA weight ratio, 35% C12-200, 16% DOPE, 46.5% cholesterol, and 2.5% C14-PEG2000. The average efficacy with 15 µg total EPO mRNA injection in vivo was obtained at 7065 ± 513 ng/µL. The particle size of optimal LNP was 102 nm, PDI: 0.158, encapsulation efficiency: 43%, pKa: 6.9, and zeta potential: −5.0 mV.

Next, Thanki et al. [15] tested the lipidoids as the lipid component of siRNA-loaded lipid-polymer hybrid nanoparticles (LPNs). They checked whether replacing the cationic lipids with lipidoids improved safety and was more efficacious (efficient gene silencing at lower doses). The initial experiments of one factor at a time were performed to identify criticality influencing the overall quality attributes of the LPNs: the lipidoid content and siRNA: lipidoid ratio. A 17-run design of an experiment with an I-optimal approach was performed to systematically assess the effect of lipidoid content % (in the range of 10–20) and weight ratio of lipidoid:siRNA (from 10:1 to 20:1) on physicochemical properties (hydrodynamic size, zeta potential, and siRNA encapsulation/loading) and the biological performance (in vitro gene silencing and cell viability). The response surface methodology was applied to the identification of optimal operating space (OOS). The optimal lipidoid-modified LPNs showed more than 50 times higher in vitro gene silencing at well-tolerated doses and about twice the increase in siRNA loading than the LPNs with cationic lipid dioleyltrimethylammonium propane (DOTAP). Another work by Thanki and co-authors [16] described the optimization of LPNs composition prepared of cationic lipidoid (L5) and poly(DL-lactic-co-glycolic acid) (PLGA) or delivery of an antisense oligonucleotide (ASO) mediating splice correction of a luciferase gene transcript (Luc-ASO). Critical formulation variables and their levels were identified in one-factor-at-a-time (OFAT) experiments. Multilevel factorial design (25 experiments) was performed for critical, independent variables with three levels: L5 content (10–20% (*w*/*w*)), L5:Luc-ASO weight ratio (in the range of 10:1 to 30:1), and molecular weight of PLGA (10–50 kDa). The following output variables were considered: Z-average, PDI, zeta potential, encapsulation efficiency, loading, and biological responses (i.e., in vitro splice correction efficiency and cell viability). Quadratic and an I-optimal model were performed, and the obtained responses were subjected to model fitting using analysis of variance (ANOVA). The best model was selected on the basis of statistical analysis. The process validation was performed by preparing and testing the five formulations with composition within the design space (range of 14–17% (*w*/*w*) L5 content and L5:Luc-ASO ratios from 11:1 to 21:1).

In turn, Blakney et al. [38] presented the optimization of self-amplifying mRNA (saRNA) lipid nanoparticle composition in human skin explants. Full factorial design was performed for five input variables: complexing lipid type (C12-200 (ionizable), dimethyldioctadecylammonium bromide [(DDA), cationic], 1,2-dioleoyl-3-trimethylammonium-propane [(DOTAP), cationic] and cephalin (zwitterionic)), total lipid to RNA ratio (*w*/*w*) (1:1, 4:1, 18:1, 90:1), lipid concentration (high (7.5 mg/mL), medium, low), particle concentration (high 10^9^, medium 10^8^, low 10^7^ particles/mL), and cationic lipid to zwitterionic lipid ratio (0.1:1, 1:1, 10:1). The luciferase expression in human skin explants after 10 days was the output variable. Cephalin lipid had the strongest effect. Cephalin LNPs with a ratio of total lipids to RNA of 18:1 (*w*/*w*), low lipid concentration, and medium particle concentration yielded a 7-fold increase in luciferase expression over the original formulation (with C12-200 lipid).

In the next paper, Hashiba et al. [39] described the optimization of liver-targeted mRNA-loaded LNPs prepared with pH-sensitive cationic lipids that had been previously designed for siRNA delivery. The first 3^4^ × 2^2^ definitive screening design (DSD, 18 formulations) was performed (library A). The input variables were qualitative factors: cationic lipid (CL) type (CL4H6, CL15H6) and phospholipid (PL) type (DSPC, DOPE) and quantitative factors: mRNA: lipid ratio (18.3–36.7), mol% of CL (40–60), mol% of PL (5–25), and mol% of PEG-DMG (0.5–2.5). The second step, a 3^4^ × 2^1^ Taguchi fractional factorial design (FFD, 18 formulations), was performed (Library B). The input variables were the qualitative factor phospholipid (PL) type (DSPC, ESM—egg sphingomyelin) and quantitative factors: mol% of CL (50–70), mol% of PL (5–15), and mol% of PEG-DMG (0.5–2.5). The cationic lipid (CL) type was fixed as a CL4H6, and the mRNA/lipid ratio was fixed as 18.3. Output variables were gene expression in the liver (Nluc expression was measured 24 h after the injection), physicochemical properties (LNP diameter and polydispersity index (PdI)), and liver-specificity (gene expression in off-target organs (spleen, lung, and kidney)). Detailed results are described in Supplementary Table S1.

The work of Lokras et al. [17] presented an approach to optimization of the intracellular delivery of therapeutic anti-inflammatory TNF-a siRNA-loaded LNPs to activated macrophages. The formulation design space was defined based on the I-optimal design for three independent formulation batches and three technical replicates. Input variables were L5 content (15; 20; 25% (*w*/*w*)) and L5:TNF-a siRNA weight ratio (5.0:1; 7.5:1; 10.0:1; 15.0:1). Output variables were PDI, zeta potential (mV), encapsulation efficiency (%), TNF-a siRNA loading (μg siRNA/mg LPNs), and fold reduction in IC50 value for TNF-a gene silencing. The optimal operating space was defined: L5 content of 15%, L5:TNF-a siRNA weight ratio of 13.5:1, and 25%, 15.0:1 respectively.

The next paper, published by Zheng et al. [18], deals with the optimization of lipid-like nanoparticles (LLNs) on the expression of luciferase mRNA in the liver containing three new cholesterol derivatives Chol-PEG400-self peptide (Chol-PEG400-SP), Chol-PEG400-Mannose (Chol-PEG400-Man), and Chol-PEG2000-W5R4K (Chol-PEG2000-WRK)) to achieve the liver-targeting delivery of mRNA. The central composite design (CCD) model (20 experiments), a multi-factor, five-level experimental design, which is formed by adding extreme points and center points based on a two-level factorial design, was investigated. Input variables were the molar ratio of the Chol-PEG400-SP (from 1.0 to 7.5%), Chol-PEG400-Man (from 1.0 to 10.0%), and Chol-PEG2000-WRK (from 1.0 to 7.5%). The output variable was in vivo Luc expression of livers measured by the IVIS (Balb/c mice i.v. injection)—bioluminescence.

Van de Berg and co-authors [19], in turn, described the bioprocess model development of rapid RNA vaccine production against emerging infectious diseases. The model parameters were fitted to a subset of 51 experimental samples from a statistical DoE dataset obtained from lab-scale saRNA synthesis experiments using wild-type nucleotide triphosphate (NTP). Thirty-three samples correspond to the RNA yield at 0.04 M NTP and 1 × 10^−8^ M of T7 RNA Polymerase (T7RNAP) vs. 11 concentrations of Mg ranging from 0.025 to 0.125 M after 2, 4, and 6 h. Moreover, 12 samples correspond to the RNA yield at 0.04 M NTP and 0.075 M Mg for 1.25 × 10^−9^, 2.5 × 10^−9^, 5 × 10^−9^, and 1 × 10^−8^ M of T7RNAP after 2, 4, and 6 h. six samples correspond to the RNA yield after 2 h at 0.02, 0.04, and 0.08 M NTP at 0.075 and 0.14 M Mg. Optimal values of input variables were NTP concentration 40.8 mM (optimization range 0.01–0.06M), T7RNAP concentration 1.5 × 10^−8^ M (optimization range 0.5 × 10^−8^–1.5 × 10^−8^), Mg concentration 85 mM (optimization 0.01–0.09 M), and reaction time: after 2, 4, and 6 h. The optimal output variables were the yield in a bioreactor: 4.34 g × L^−1^, and the cost of T7RNAP and NTPs per g of RNA: 2740 USD × g^−1^.

In the paper by Fan et al. [20], the optimization of LNPs loaded with antisense oligonucleotides (ASOs), formulated by an automated solvent-injection method using a robotic liquid handler (e.g., muscular dystrophy Duchenne’s) was performed. In the first step, optimization of the phase mixing process using a robotic TECAN liquid handler was carried out. Input variables were investigated as an injection speed: 0.1, 0.5, or 0.9 mL/s at 10 mixing repeats, and the ethanol-to-buffer injection at a speed of 0.5 or 0.9 mL/s followed by 10 or 20 mixing repeats. Output variables were particle size, polydispersity, and encapsulation efficiency (calculated based on free ASO-1 measured by OD260). The second step involved the high throughput screening (HTS) workflow for ASO-loaded LNP formulations (96 samples). Input variables were two levels of total lipid concentrations (1 mM; 2 mM), four levels of ASO loading controlled by N/P ratios (0.5; 1; 2; 5), and four levels of the PEGylated lipid (DSPE-PEG2000) content (0; 1.5; 3; 5 %mol of total 2 mM lipids). Output variables were particle size (obtained in the range of 45–145 nm), polydispersity %PD (10–50), and ASO encapsulation efficiency measured by absorbance at 260 nm. We observed that 5 mol% of DSPE-PEG2000 and an N/P ratio ≥ 1 would produce optimal LNP formulations with a homogeneous and stable particle size as well as high ASO loading. Other detailed results are summarized in Table S1.

Terada et al. [40] described the DOE approach to find the preparation conditions and their relationship with the properties of LNP-siRNA. The design included three levels of the three-factor Box–Behnken design: low, center, and high, and three center samples (15 preparation). The influence of the input variables: lipid concentration (10, 20, 30 mM), flow rate ratio (FRR) (1, 3, 5 (*v*/*v*)), and total flow rate (TFR) (1, 2, 3 mL/min) on the output parameters: the particle size, polydispersity index (PDI), and siRNA entrapment, was tested. The charge ratio (N/P) in the prepared LNP-siRNA systems was fixed at 3. LNPs were prepared using hydrogenated soy phosphatidylcholine (HSPC), cholesterol (Chol), and 1,2-distearoyl-sn-glycero-3-phosphoethanol amine-*N*-[methoxy-(polyethylene glycol)-2000] (ammonium salt) (PEG-DSPE). In addition, 1,2-dioleoyl-3-dimethylammonium propane (DODAP) was used which has a reported apparent pKa of 6.5. The optimal lipid composition was DODAP/Chol/HSPC/PEG-DSPE (50/10/39/1 %mol).

The next authors, Bevers et al. [21], optimized mRNA-LNP compositions to achieve high-magnitude tumor-specific CD8 T cell antitumor immunotherapy by engaging the splenic immune cells. A Roquemore hybrid design was used to select the formulations to be tested (11 different experimental conditions). Each condition was applied in triplicate (3 mice /treatment), and each lipid’s molar percentage takes five values. The experimental protocol was carried out for three different choices of PEG-lipid chemistry (DMG-PEG2000, DSG-PEG2000, and DSPE-PEG1000) and different lipid molar ratios (10, 20, and 30 mM). The highest %E7-specfic T cell response was investigated as an output variable. The optimal LNPs composition were: DMG-PEG2000: ionizable lipid 56.5%, DOPE 5.25%, cholesterol 37.75%, PEG-lipid 0.5%, and DSG-PEG2000: ionizable lipid 64.4%, DOPE 8%, cholesterol 27.1%, PEG-lipid 0.5%. More detailed relationships between variables are presented in Table S1. 

Karl et al. [22], in turn, proposed a holistic workflow for lipid nanoparticle (LNP) formulation optimization for in vivo gene delivery systems. They used designed mixture-process experiments and a self-validated ensemble model (SVEM) (23 runs, LNP batches were tested). The generally used input variables were listed and presented on the fishbone diagram, e.g., ionizable lipid type (H101, H102, H103), ionizable lipid molar content (10–60%), helper lipid type, helper lipid molar content (10–60%), PEG lipid type, PEG lipid molar content (10–50%), N/P ratio (6–14), buffer concentration, and flow rate (1–3 mL/min). Output variables were maximum potency, minimum particle size, minimum PDI (polydispersity index), maximum % encapsulation, and minimum side effects—such as body weight loss in in vivo studies. Exemplary optimal LNP formulation candidates were described in Table S1.

The paper by Schmidt et al. [23] refers to a general approach to process automation and control strategy by QbD in total continuous mRNA manufacturing platforms (mRNA)-based vaccines. The work contains very detailed information about all aspects of LNP-mRNA formation, process parameters, and product composition based on risk analysis. The authors presented, e.g., Ishikawa diagrams to identify all possible sources of variability. Different multivariate optimization prediction was comparable: Ordinary least squares (OLS) regression, partial least squares (PLS) regression, and neural network (NN) regression. Full factorial design and one factor at a time (OFAT) analysis were also used. The optimal process and product parameters are listed in Table S1.

In the next paper, Toma et al. [24] presented a general QbD approach for the development of miRNA nonviral vectors for genetic material delivery in cancer therapy. The DOE is defined generally as providing better results with a minimum number of experiments and evaluating CMAs and CPPs to obtain a product meeting the QTPP. 

Young and co-authors [25] described that optimization of LNP composition drives the delivery of mRNA to the placenta. In the DOE approach, a factorial design study was performed. The definitive screening design (DSD) was used to create a mini-library of 18 chemically unique LNPs (A1–A18). A combination of three-level continuous and two-level categorical factors to identify linear and quadratic effects was used: ionizable lipid type (C12-200, DLin-MC3-DMA), phospholipid type (DSPC, DOPE), ionizable lipid molar percentage (25–45%), phospholipid molar percentage (10–22%), DMPE-PEG molar percentage (1.5–3.5%), and cholesterol molar percentage (add up to 100%). Output variables were hydrodynamic diameter (obtained in the range of 72.2–171.5 nm), polydispersity index (0.120–0.317), mRNA encapsulation efficiency (35.6–83.2%), transfection efficiency, and apparent pKa (TNS assay) (5.298 to 7.111). Optimal LNP was formulation A10 with the following composition and characterization: 35% C12-200, 10% DOPE, 1.5% PEG, 53.5% cholesterol; 130.2 nm hydrodynamic diameter, 0.064 PDI, 56.5% EE, 6.607 pKa.

The next paper, by Nag et al. [26], presented a novel approach to size regulation of LNPs using the combined effects of buffer exchanger pH and sonication time. Input variables were: pH of buffer exchanger (4.0–5.0), sonication time (0–100 s), buffer dialysis type (50 mM HEPES pH 6.7, 50 mM HEPES/50 mM sodium acetate pH 6.7, PBS pH 7.2, PBS pH 7.4), and % of dialysis buffer (75–100%). Optimized parameters were LNP size (nm) and polydispersity index (PDI). Optimal values of LNP diameter were 60–180 nm (±10 nm) and PDI ≤ 0.200.

DOE approach to the optimization of lipid nanoparticles for self-amplifying mRNA (saRNA) expression and cellular activation was presented by Ly et al. [27]. The first optimization was performed based on a definitive screening design (DSD). A 7 three-level matrix was built with two two-level qualitative factors and one three-level qualitative factor (iteration A, 26 formulations). Input variables were: N/P ratio (5; 10; 15), phospholipid type: DOPE; DSPC phospholipid content (10; 15; 20 mol %), ionizable lipid type (pKa) (DLin-MC3-DMA (6.4); ALC-0315 (6.09); SM-102 (6.75)), ionizable lipid content (30; 40; 50 %mol), DMG-PEG-2000 content (0; 1.25; 2.5 %mol) total flow rate during preparation (2; 9; 16 mL/min), ambient temperature during formulation (4; 20; 37 °C), aqueous-phase pH (3; 5; 7), and RNA type (mRNA; saRNA). Output variables were size, PDI, EE%, charge, % filled particles, and % full RNA transcripts. Apart from standard statistical methods such as variance analysis (ANOVA), linear regression, and Spearman’s correlation, Python’s scikit-learn module, statsmodels package, and the seaborn library were also used. The second optimization was performed based on a Box–Behnken design (BBD). Three three-level and 1 three-level quantitative factor (iteration B, 26 formulation) were tested. Input variables were phospholipid content (12.5; 15; 17.5 %mol), ionizable lipid type (pKa) (DLin-MC3-DMA (6.4); ALC-0315 (6.09); SM-102 (6.75)), ionizable lipid content (35; 40; 45 %mol), and aqueous-phase pH (4; 5; 6). The N/P ratio was fixed at 10, DOPE was used as a phospholipid, DMG-PEG-2000 content was fixed at 1.25 %mol, the total flow rate was fixed at 16 mL/min, and ambient temperature during formulation was fixed at 20 °C). Output variables were size, PDI, EE%, charge, % filled particles, % full RNA transcripts, protein expression, and cellular activation. Response surface modeling was carried out using second-order ordinary least square (OLS) regression based on the scikit-learn module and statsmodels package. A Box–Cox transformation of response variables was used to improve model accuracy. The optimization of simultaneous responses was realized by the desirability function, and optimal operating conditions were obtained using the SciPy 1.0 library and Broyden–Fletcher–Goldfarb–Shanno (BFGS) optimization algorithm. 

The paper by Mendonca et al. [28] presented a review of LNP delivery of different types of nucleic acids: siRNA, mRNA, and pDNA. Stricted DOE was not described, but a lot of important variables that affected the effectiveness of LNP formulations were listed (Table S1).

The last presented work by Bastogne et al. [29] concerns the optimization of cationic nano-lipid for siRNA transfection. DOE was performed based on a D-optimal mixture design (36 formulations were tested). Input variables were DOTAP proportion (%) in the LNP content, the concentration of PEG surfactant (%), the lecithin proportion (%), and LNP size (small, medium, large). Output variables were safety attributes related to the LNP stability (unstable or stable states, 0 or 1), siRNA transfection rate (PC3-GFP) (min 30%), and PDI. Response surface equations, a class of polynomial models, are used to describe the links between the input and output variables. Bayesian estimation method, the posterior predictive check, and the leave-one-out cross-correlation were used to evaluate the model prediction.

## 4. Discussion

Optimization of lipid-based RNA formulations is essential to achieve reproducible quality of pharmaceutical products in terms of efficacy and safety; therefore, the application of QbD and DOE appears to be a very useful tool for the robust development of this complex RNA-LNP system. The performance of LNPs is strongly influenced by the chemical structure of each component, the interactions between them, and the physicochemical properties of the final formulation.

The most important characteristics of RNA-LNPs are zeta potential, size and particle size distribution, shape, morphology, encapsulation efficiency, cellular uptake, and transfection efficiency. The chemical structure of lipids, lipid concentration, and lipid molar ratio were the most significant factors regulating formulation average and gene expression [15,16,28,38,39]. Cationic lipids for gene transfer have become a major research tool for the transfer of genetic material into cells, and there is great potential for progress in this direction [20].

Particle size was retained as one parameter of QTPP as it is widely known to affect pharmacokinetics, tissue distribution, tissue extravasation, uptake and/or accumulation in clearance organs [15]. The particle size of formulation LNPs depends most on the composition of lipids. In general, the presence of PEG improved particle stability and reduced size but hindered delivery efficiency [27,29,36,37]. Particle size and PDI decrease with increasing RNA: ionizable lipid ratio due to higher RNA concentrations and concomitantly reduced amount of ionizable lipid [15,16].

Another parameter characteristic of RNA-LNP is the zeta potential, which also depends on the lipid type and concentration. In general, QTPP for zeta potential was set to >0 mV because nanoparticles with positive zeta potential enhanced interactions with the plasma membrane. Positively charged nanoparticles can encapsulate negatively charged genetic material by electrostatic interaction [24]. Depending on the composition and especially the modifications of the end groups (acid, amine, or esters), LNP particles can have a negative [20,22,25] or a positive charge [15,17,24]. Zeta potential increased proportionally as a function of ionizable lipid content and RNA: ionizable lipid ratio [15,16,17]. Zeta potential showed a significant correlation with transfection efficiency [27,28,36].

Another important CQA is entrapment efficiency, measured as an encapsulation efficiency. Encapsulation efficiency is more dependent on the LPN preparation process rather than independent variables [17,26]. No statistically significant differences in encapsulation efficiency were observed with particle size and PDI [15] but increased with higher RNA: ionizable lipid ratio [15,17,27,37].

All of the above particle properties contribute to biological efficacy but are not always the same in vitro and in vivo studies. For example, the efficacy of lipoplex increased with increasing hydrocarbon chain length, whereas the in vitro efficacy of LNP did not change significantly [35]. In vivo, the efficacy of LNP decreased with increasing hydrophobicity of cationic lipids [35]. The presence of the phospholipid DOPE was generally the strongest predictor of in vivo efficacy [36,37,39]. The specific combination of ionizable lipid and phospholipid in the LNP design provides high transfection efficiency in vitro [25].

The DOE approach has been used in most of the optimization works; however, the full QbD approach has been described in only a few papers [19,23,24,25,28], and a few articles refer only to some aspects of QbD—the definition of QTPP and CQA [15,16,17,20,27].

We have seen different approaches and innovations in DOE and statistical analysis. Traditional statistical tests and modeling based on ANOVA and regression analysis are slowly being replaced by artificial intelligence and machine learning methods, e.g., neural networks [23]. From a methodological point of view, the most interesting new development in pharmacy is the development of self-validated ensemble models (SVEM), especially with the connection of mixture design [22].

## 5. Conclusions

Over the past 10 years, many research articles have described the development of studies of RNA-loaded lipid nanoparticles for various clinical purposes, e.g., prophylaxis of infectious diseases, treatment of rare diseases, and gene, cancer, and protein replacement therapy. In addition, the coronavirus disease 2019 (COVID-19) pandemic and the emergence of safe and effective RNA vaccines have brought RNA technology to the forefront of medical innovation. Due to the mechanism of action, the therapeutic scope of RNA technology is wide, and the manufacturing processes are versatile. Different products could be produced using the same raw materials, consumables, equipment, unit operations, and analytical methods. In parallel with the development of new drug delivery methods, the QbD approach is becoming increasingly widespread in pharmaceutical manufacturing. 

This review could be very useful for researchers and pharmaceutical manufacturers to apply the quality by design approach to the development of lipid nanosystems loaded with different types of RNA, following the latest trends and regulatory requirements and using modern mathematical and statistical design methods.

## Figures and Tables

**Table 1:** Optimization of RNA-LNP system information about optimizing variables and the tested range was described in the Section DOE Approach—Design Models, Variables, and Range.

References	The Main Goal/Target/Delivery System	QbD Aspects	DOE/Mathematical Model/Statistical Analysis Tests and Software
[34]	siRNA-loaded PLGA poly(DL-lactide-glycolide acid) without cationic excipients prepared by the double emulsion solvent evaporation method	Not applied	2^(5−1)^ fractional factorial design (FFD) with centre point
[35]	siRNA-containing lipid nanoparticles (LNPs) prepared by microfluidic method	Not applied	High-throughput synthesis and screening Correlation analysis of the structure–function relationships
[36]	mRNA TT (*N*1,*N*3,*N*5-tris(2-aminoethyl)benzene-1,3,5-tri carboxamide) lipid-like nanoparticles (TT-LLNs)	Not applied	Design of N1,N3,N5-(TT) derivatives Correlations analysis Orthogonal experimental design L16(44)
[37]	Erythropoietin(EPO)-mRNA-loaded C12-200 lipid nanoparticles	Not applied	Library A: Definitive screening design (economical design of experiment), factorial design (14 experiments) Library B: Taguchi fractional factorial screening design (18 experiments) Standard least squares linear regression model, ANOVA, posthoc Tukey test to verify phospholipid effect, Student’s *t*-test Library C: Maximizing lipid:mRNA weight ratio with DOPE (6 experiments)
[15]	siRNA-loaded lipid-polymer hybrid nanoparticles (LPNs)	Critical quality attributes (CQAs): - hydrodynamic size - zeta potential - siRNA encapsulation/loading - in vitro gene silencing - cell viability Quality target product profile (QTPP) of optimized LPNs: - z-average < 250 nm - polydispersity index (PDI) < 0.3 - zeta potential > 0 mV - encapsulation efficiency > 60% - siRNA loading efficiency > 5 μg/mg - transfection efficiency (IC50) < 5 nM - cell cytotoxicity (IC50) > 50 nM OOS (optimal operating space)	The initial experiments: one factor at a time to identify criticality influencing the overall quality attributes of the LPNs 32 factorial designs with eight augmented points (17 experiments) Quadratic model and I-optimal model The response surface methodology (RSM)—OOS (optimal operating space) identification
[16]	LPNs composed of cationic lipidoid 5 (L5) and poly(DL-lactic-co-glycolic acid) (PLGA) or delivery of an ASO mediating splice correction of a luciferase gene transcript (Luc-ASO)	The quality target product profile (QTPP): safety and efficacy Critical quality attributes (CQAs): - z-average - PDI - zeta potential - Luc-ASO encapsulation efficiency - Luc-ASO loading - in vitro splice-correction efficiency - in vitro cell viability. Z-average < 250 nm is optimal for intracellular delivery: rapid cellular internalization with sustained release PDI < 0.3: narrow particle size distribution and colloidal stability Zeta potential of the LPNs < 30 mV: minimized cellular toxicity Encapsulation efficiency > 70%: reduced cost of goods (pharmacoeconomic consideration) Loading of Luc-ASO > 6 μg/mg: reduced effective particle dose The slope for splice correction was set to >0.01: reduced effective ASO dose and/or improved therapeutic efficacy Luc-ASO IC50 value > 150 nM: reduced effective ASO dose and/or improved therapeutic efficacy	Critical formulation variables and their levels were identified in one-factor-at-a-time (OFAT) experiments Multilevel factorial design (25 experiments), three levels of two critical independent variables Statistical analysis: - Quadratic and an I-optimal model - The obtained responses were subjected to model fitting using analysis of variance (ANOVA), and the best model fit was selected based on statistical parameters - The statistical data treatment was performed using the Design Expert software (version 11, statease, Minneapolis, MN, USA) - The data were further treated numerically and graphically to generate desirability and overlay plots, respectively - Five formulations were selected from the resulting design space, and they were prepared and characterized for validation purposes
[38]	saRNA lipid nanoparticles in human skin explants	Not applied	Design of Experiment and Statistical Analysis: - Full factorial design - Jmp, version 13.0, - Standard least-squares for effect screening with the model effects designated as first- and second-order effects only - Nonsignificant effects were excluded from the model Graphs prepared in GraphPad Prism software, version 7.0. Flow cytometry statistical analysis performed in Prism software, using a two-tailed *t*-test with α = 0.05, which was used to indicate significance
[39]	Liver-targeted mRNA-loaded LNPs prepared with pH-sensitive cationic lipids that had been previously designed for siRNA delivery	Not applied	DOE with multiple responses Library A (18 formulations): 3^4^ × 2^2^ definitive screening design (DSD) Library B (18 formulations): 3^4^ × 2^1^ L18-Taguchi fractional factorial design (FFD) Characterization of the optimized mRNA-loaded LNPs Statistical analysis: - Least squares linear regression model was applied to each response - Effective design-based model selection for DSD or the forward stepwise regression method with Akaike’s information criterion and finite correction (c-AIC) was applied to each response (where the number of both statistically significant main factors and interactions between 2 factors were less than 3). - Comparisons between the means of two variables, unpaired Student’s *t*-tests were used, one-way ANOVA with the Student–Newman–Keuls post hoc test was used for comparisons between multiple groups - DOE - Was analyzed using the JMP 14 software (SAS, Cary, NC, USA) - Statistical significance was defined as *p*-values less than 0.05. - Tree mice per formulation were used for the DOE - Graphs prepared in GraphPad Prism software, version 7.0.
[17]	Intracellular delivery of therapeutic anti-inflammatory TNF-a siRNA loaded LNPs to activated macrophages	The quality target product profile (QTPP): - Efficient and safe - Gene silencing mediated by TNF-a siRNA-loaded LPNs in vitro in the murine macrophage cell line RAW264.7 - Loading a higher dose of siRNA in the LPNs does not correlate with higher gene silencing in vitro Critical quality attributes (CQAs): - Z-average (maximal uptake by macrophages): <200 nm - PDI (predictable particle behavior): <0.200 Zeta potential (enhanced interaction with macrophages): >0.0 mV - Encapsulation efficiency (pharmacoeconomic consideration): >60.0% - Loading efficiency (reduced effective dose of siRNA and delivery system): > 6.0μg/mg LPNs - Gene silencing efficiency (IC50): <20.0 nM - Cell viability: >200 nM	Formulation design space: I-optimal design, three independent formulation batches, and three technical replicates (*N* = 3 and *n* = 3) Statistical Analysis: - Data were analyzed using GraphPad Prism Software version 8 - Results presented as mean values ± standard deviation (SD) - Statistically significant differences were assessed by one-way analysis of variance (ANOVA) followed by Tukey’s post-hoc multiple comparison test - *p*-value ≤ 0.05 was considered statistically significant
[18]	Lipid-like nanoparticles (O-LLNs) containing three new cholesterol derivatives to achieve the liver-targeting delivery of mRNA. The O-LLNs outperformed DLin-MC3-DMA (MC3) in the functional delivery of Cre-recombinase (Cre) and human erythropoietin (hEPO) mRNA. Delivery of cytidine base editor mRNA (CBE mRNA) and sgRNA by O-LLNs in a liver-related metabolism disorder, phenylketonuria (PKU).	QbD not applied LLNs characterization: - mRNA loaded into the hydrophilic core with a G0-C14 (ionizable lipid)-to-mRNA mass ratio of 7.5/1. - Particle size: from 125 to 137 nm - Polydispersity index (PDI) < 0.3 - Zeta potential from 33 to 45 mV - The encapsulation efficiency is about 70% - 30 μg of Luc mRNA	Optimization of LLNs on the expression of luciferase mRNA in livers CCD (central composite design) Model (20 experiments): - Multi-factor five-level experimental design, which is formed by adding extreme points and center points based on a two-level factorial design Statistical Analysis: - Expert Design software (DOE) - Graph pad Prism 7.0. - Second-order polynomial model - All values were expressed as mean ± S.D. unless otherwise noted - Data from more than two groups were compared using one-way analysis of variance (ANOVA), and multiple comparisons were performed by Tukey’s post-hoc test when determined significant - *p*-values less than 0.05 were considered statistically significant
[19]	Rapid RNA vaccine production against emerging infectious diseases	The quality target product profile (QTPP): - Product safety and efficacy Critical quality attributes (CQAs): - Rank and identify CQAs of the mRNA and siRNA vaccine based on their impact and uncertainty scores for both product safety and efficacy - RNA yield, - Sequence integrity, - Sequence identity - 5′ capping efficiency Critical process parameters (CPPs) - Temperature - Reaction time - pH in transcription reactor - DNA template sequence - DNA template concentration - T7RNAP concentration - 5′ cap analogue concentration - Total Mg concentration - DTT concentration - Spermidine concentration - GTP concentration - Total NTP concentration - Ratio of NTPs	Bioprocess modeldevelopment: - The model parameters were then fitted to a subset of 51 experimental samples from a statistical DoE dataset obtained from lab-scale saRNA synthesis experiments using wild-type NTPs Statistical analysis: - MODDE® statistical Design of Experiments software - Two separate models were fitted using MLR, one using the four factors as linear predictors and one that also included square terms in the Mg and NTP concentrations as well as an interaction term consisting of the product of Mg and NTP concentrations
[20]	LNPs loaded with antisense oligonucleotides (ASOs) formulated by an automated solvent-injection method using a robotic liquid handler (e.g., Duchenne muscular dystrophy)	Critical quality attributes (CQAs): - Particle structure - Size distribution - Physicochemical properties of the particle surface - Lipid content - Amount of the free API and encapsulation efficiency - Physical and chemical stability Critical process parameters (CPPs) - Microfluidic manufacturing of LNP formulations: total flow rates and flow rate ratios are critical to producing small and homogenous particles - While different N/P ratios and buffers determine LNP formation mechanisms and their different particle structures	Optimization of the phase mixing process using a robotic TECAN liquid handler HTS workflow for ASO-loaded LNP formulations (96 samples, *n* = 3) Validation of HTS results with scale-up LNP preparation—comparing results from the HTS approach with those from a microfluidic formulator Statistical Analysis: - All results are presented as mean ± SD, *n* = 3 - Data were analyzed by one-way or two-way analysis of variance (ANOVA) followed by Turkey’s, Sidak’s, or Dunnett’s post-tests for comparison of multiple groups - GraphPad Software (Prism 8.0) - *p*-values less than 0.05 were considered statistically significant
[40]	LNP-siRNA systems as a promising approach for silencing disease-causing genes in hepatocytes following intravenous administration	Not applied	DOE approach: - The three-factor Box–Behnken design, three levels: low, center, and high, and three center samples were included in this design as a source for error estimation (15 preparation) - Quadratic model The relationship between the preparation parameters and the properties was evaluated for LNPs with and without siRNA by performing the same procedure for empty LNPs Statistical analysis: - All values were indicated as mean ± standard deviation. Statistical comparisons between two conditions were performed using paired Student’s *t*-test. Values of *p* < 0.05 were considered to indicate statistical significance - The experimental data were analyzed with the statistical software JMP 13 (SAS Institute)
[21]	mRNA-LNP compositions to achieve high-magnitude tumor-specific CD8 T cell antitumor immunotherapy by engaging splenic immune cells	Critical quality attributes (CQAs): - CD8T cell response, determined as %E7-specific T cells in blood of mice after three immunizations > 50% Critical material attributes (CMAs): - Ionizable lipid content: 36–65% - PEG-lipid content: 0.5–2% - DOPE content: 5–15% - Cholesterol content: fill molar proportion up to 100% Design space (DS) - NOR (normal operating region): probability to respect the specification of at least 50% E7-specific T cells greater than 90% - PAR (proven acceptance region): probability to respect the specification between 70% and 90% - OOS (out of specification): probability lower than 70% to respect the specification	A Roquemore’s hybrid design was used to select the formulations to be tested - 11 different experimental conditions - Each condition was applied in triplicate (3 mice/treatment) - Each lipid’s molar percentage takes five values - Experimental protocol was carried out for three different choices of PEG-lipid Statistical Analysis: - Quadratic response surface model - The Hamiltonian Monte Carlo (HMC) algorithm (a generalization of the Metropolis algorithm, a family of Markov chain Monte Carlo (MCMC) algorithms) was used to compute the posterior distributions of the model parameters. Simulations from the HMC are determined by Bayes’ rule, in which the posterior distribution of the parameters is proportional to the prior distribution of the parameters multiplied by the likelihood. - The posterior predictive check (PPC) is a Bayesian technique to assess the appropriateness of the model to fit data - Sensitivity indices of the %E7-specific T cells for the three lipid proportions were computed based on a Monte Carlo estimation of the Sobol’s indices - GraphPad Prism software 8.0 and 9.0 were used for the statistical analyses indicated in figure legends - Significant differences are indicated as *p* < 0.05.
[22]	Lipid nanoparticle (LNP) formulations for vivo gene delivery systems	Summarizes a quality by design (QbD) styled approach to the optimization of lipid nanoparticle (LNP) formulations	Self-validated ensemble model (SVEM) (23 runs, LNP batches): Statistical Analysis: - Self-validated ensemble model (SVEM)—has greatly improved the approach to analyzing results from mixture-process experiments - JMP Pro 17.1 with SVEM, along with the graphical summary tools
[23]	Process Automation and Control Strategy by QbD in Total Continuous mRNA Manufacturing Platforms (mRNA)-based vaccines	Process analytical technology (PAT) and process control strategies Critical process/product parameters (CPPs): - In vitro Transcription: space-time yield (STY) of capped mRNA - Inline Diafiltration by SPTFF (single-pass tangential flow filtration): pressure drop, fouling, low concentration-factor - LNP formation: efficacy/bioavailability Risk assessment and impact ranking—critical quality attributes (CQAs) defined - Fish Bone/Ishikawa diagram/one factor at a time (OFAT) - In vitro Transcription: NTP concentration, cap analog concentration, temperature, pH, variation flow, length to diameter - Inline diafiltration by SPTFF: variation of flow feed, viscosity, fiber diameter, fiber length, fiber count, Rm, TMB, variation flow EB - LNP formation: mRNA conc, mRNA Feed pH, lipids:mRNA weight ratio, w% ionizable lipid, *w*/*w* ionizable/PEG-Lipid, *w*/*w* ionizable/help Lipid, *w*/*w* ionizable/Cholesterol, volume flow rate, *v*/*v* aq/EtOH, diafiltration volumes Multivariate studies and design space characterization—based on DOE studies Process Control Strategies - In vitro transcription: pH—decrease due to reaction +0.1/−1, temperature—ambient temperature change ±0.5 °C, mass flow—deviating pump speed ±5%, master mix—deviating concentration(s) ±10% - Inline diafiltration by SPTFF: mRNA—deviating concentration ±10%, flow rate feed—deviating pump speed ±5%, flow rate EB—deviating pump speed ±5%, permeate flux decrease due to fouling ±10% - LNP formation: mRNA—deviating concentration ±10%, pH aq. buffer—deviating value ±0.2, lipids—deviating concentration(s) ±5%, flow rate—deviating pump speed ±5%, Dv—deviating pump speed ±5%, permeate flux—decrease due to fouling ±10%	DOE approach, Statistical Analysis - Full-factorial design - Ordinary least squares (OLS) regression - Partial least squares (PLS) regression - Neural network (NN) regression
[24]	miRNA nonviral vectors for genetic material delivery in cancer therapy	Quality Target Product Profile (QTPP) of Nonviral Vectors: - Efficacy and safety - Administration route: intravenous—to improve the efficacy and bioavailability; direct availability in the bloodstream - Dosage form: injection—low volume production allows customization to client/quantities - Delivery system element: nonviral vector—provides safer and more effective delivery of the genetic material - Ph: 7.35–7.45—to prevent or reduce vascular complications - Osmolarity: 290–310 mOsm/L—to ensure tolerability - Particle size: below 200 nm—to ensure penetration in the cell - Homogeneity: monodisperse—to ensure the system’s homogeneity - Enhanced therapeutic activity: high transfection efficiency (over 80%)—to improve the system’s effectiveness - Storage condition −60° - C ± 20 °C—to guarantee the stability of the genetic material - Improved safety: lack of cytotoxicity, lack of hemolytic activity—to ensure appropriate biological requirements - Microbiological quality: sterile and pyrogen free—to avoid contamination with microorganisms; to ensure patient safety - In vitro release: prolonged release—to ensure release according to a predefined release pattern or to ensure spatiotemporal release of the payload Critical quality attributes (CQAs) for nonviral vector - Particle size: 100–400 nm—internalization in tumor cells - Polydispersity index (PDI): 0.1–0.5—narrow size distribution, homogeneity of the nanosystem in terms of size - Zeta potential (ZP): 5–30 mV—formation of electrostatic bonds between the vector and the cell environment - Surface modifications: hyaluronic acid, transferrin, PEG—decreased opsonization and phagocytosis, prolonged circulation - Cytotoxicity: High IC50—to ensure nanosystem safety - Cellular uptake: efficient cellular uptake—to ensure penetration in the cell - Transfection efficiency: over 80%—to ensure the desired biological effect Critical process parameters (CPPs) of nanoparticles for genetic material delivery. - Stirring speed and time, Polyelectrolyte concentration—gold nanoparticles, layer-by-layer - Stirring speed and time, ultracentrifugation speed—gold nanoparticles, laser ablation in liquid - Incubation time, temperature—liposomes, film dispersion method - Evaporation time, pressure and temperature, hydration time and temperature—liposomes, thin film hydration method - Injection rate—liposomes, ethanol injection method - Mixing speed and temperature—polymeric nanoparticles, o/w single emulsion method - Sonication time and amplitude, stirring time and temperature—polymeric nanoparticles, double-emulsion method - Sonication time, agitation time, temperature, and speed—SLN (solid lipid nanoparticle), solvent diffusion method - Sonication time—SLN, film-ultrasonic method Critical material attributes (CMAs)—physical, chemical, biological, or microbiological properties that must comply to ensure the desired CQAs - The materials used for genetic material delivery should be suitable for interaction with the human body, and their most important feature is their biodegradability - Phospholipid type - Number of hydrophobic carbon atoms in the lipid tail - Genetic material: lipids ratio or genetic material: nanoparticles ratio	The DOE’s advantages are providing better results with a minimum number of experiments and evaluating CMAs and CPPs to obtain a product meeting the QTPP
[25]	LNPs that enable high levels of mRNA delivery to trophoblasts in vitro and to the placenta in vivo with no toxicity to treat placental dysfunction	Not applied	Design of experiments (DOE) - Factorial design study - Definitive screening design (DSD) was used to create a mini-library of 18 chemically unique LNPs (A1–A18) - Combination of three-level continuous and two-level categorical factors to identify linear and quadratic effects Statistical analysis: - JMP Pro 16 (SAS Institute Inc., Cary, NC, USA) software - GraphPad Prism 9.0 (San Diego, CA, USA) software - *p*-value statistically significant level 0.05 - After active effects are identified in the combined model parameter estimates - Standard least squares fit is applied to obtain the significant effects in the fit model - All experiments have *n* = 3 replicates unless otherwise indicated - Continuous features were assessed for normality using D’Agostino–Pearson omnibus (K2), Anderson–Darling (A2), Shapiro–Wilk (W), and/or Kolmogorov–Smirnov (distance) - Results are represented as mean with standard error of the mean (SEM), and statistical significance was determined at 0.05.
[26]	Pharmaceutical-grade wide-range LNPs for RNA-vaccine/drug delivery	Critical quality attribute (CQA): - Size and size distribution of particles - Polydispersity index (PDI) ≤ 0.30	Input variables: - pH - Sonication time Output variables LNP diameter
[27]	Lipid nanoparticles for saRNA expression and cellular activation	Critical quality attribute (CQA): - Particle size (Z-average diameter) between 80 and 100 nm - Polydispersity index (PDI) less than 0.2 - Encapsulation efficiency (EE) at least 80% - Neutral zeta potential	Iteration A—definitive screening design (DSD), 7 three-level, 2 two-level qualitative factors, 1 three-level qualitative factor (26 formulations): Iteration B—Box–Behnken design (BBD); 3 three-level, 1 three-level quantitative factors (26 formulations) Statistical analysis: - Duplicates were used in iteration A; triplicates were used for iteration B - Variance (ANOVA), linear regression, and Spearman’s correlation methods - Python: the sci-kit-learn module and statsmodel package were used, and the seaborn library was used for plotting - Model statistical significance was defined as *p*-values less than 0.05, and lack-of-fit was considered insignificant at a level of 0.1 - For iteration B, response surface modeling was carried out using second-order ordinary least square (OLS) regression based on the sci-kit-learn module and statsmodel package - Box–Cox transformation of response variables did not strongly improve model accuracy - The optimization of simultaneous responses was realized by the desirability function - Optimal operating conditions were obtained using the scipy library and Broyden–Fletcher–Goldfarb–Shanno (BFGS) optimization algorithm JMP 13 software (SAS Institute)
[28]	LNPs delivery of different types of nucleic acids siRNA, mRNA, and pDNA—Review	Quality Target Product Profile (QTPP): - Intended clinical use - Administration route - Duration of activity - Pharmaceutical dosage form - Excipients - Dosage regimen - Safety/immunogenicity - Stability and storage - Efficacy - Presentation Critical quality attributes (CQAs): Physicochemical properties: - Particle size, PDI, zeta potential - Loading efficiency - Stability in biological fluids - Shelf life and storage conditions - Release of cargo Activity in cell-based assays: - Cell viability > 80% - Gene expression <50% at nucleic acid concentrations < 100 nM in serum-containing media - Less than 10% gene expression by scrambled nucleic acid at <100 nM concentration in serum-containing media Activity in in vivo studies: - Risk that an attribute impacts safety or efficacy (e.g., PK/PD, Immunogenicity, bioactivity) Critical material attributes (CMAs): - Excipients, blends, and ratios - Non-toxicity - Biodegradability Critical process parameters (CPPs): - Temperature - Microfluidics - Lyophilization	Not applied
[29]	Cationic nano-lipid for siRNA transfection	Quality target product profile (QTPP): - Intended use - Dosage form and appearance - Route of administration - Stability - Physical attributes, purity, sterility, and water content - Business information about the targeted market Critical quality attribute (CQA): - Physical, chemical, or biological property - Characteristics that must be kept within an appropriate range to ensure the desired product quality defined in the QTTP, efficacy/safety, and quality Critical manufacturing attributes (CMA): - The first category of input factors able to cause variability of CQA associated with the formulation parameters Critical process parameters (CPP): - The second category of input variables Design Space: - Identify the operating region of quality based on risk analysis - Graphical representation is used to determine the CPP values, allowing the quality requirements to be met with a controlled probability - Out of specification (OOS) region: the probabilities of meeting the technical requirements on CQA are too small - Proven acceptance region (PAR) probabilities of meeting requirements are acceptable - Normal operating region (NOR): a desired region where CQAs have a high degree of probability of complying with their quality specifications - Control operating region (COR)—subspace of NOR in which an automated control system has to maintain the operating point	Design of experiments (DOE): - Stimulate in a minimum of tests CPP and CMA - The collection of measurements on the CQA during the experiments - Better understanding the cause-effect relationship between those variables to finally predict the risks of not complying with the efficacy/safety/quality specifications Modeling: - The mathematical relationship between quality outcomes (CQA) and input factors (CMA and CPP) - Response surface equations, a class of polynomial models, are often used to describe the links between input and output variables Statistical analysis - D-optimal mixture design (36 formulations) - Design Expert Software - Bayesian estimation method - The posterior predictive check The leave-one-out cross-correlation

## Data Availability

No new data were created or analyzed in this study. Data sharing is not applicable to this article.

## Supplementary Materials

The following supporting information can be downloaded at https://www.mdpi.com/article/10.3390/biomedicines11102752/s1, Table S1: Optimization of RNA-LNP system using the QbD and/or DOE approach—summary and results.
