# Supplementary material for: Quality by Design (QbD) and Design of Experiments (DOE) as a Strategy for Tuning Lipid Nanoparticle Formulations for RNA Delivery
Source: Biomedicines. 2023 Oct 11;11(10):2752. doi: 10.3390/biomedicines11102752 (PMC10604315; doi:10.3390/biomedicines11102752)
Supplement: Supplementary file 1 [file biomedicines-11-02752-s001.zip › biomedicines-2622742-supplementary.pdf]

Table S1. Optimization of RNA-LNP system using the QbD and/or DOE approach – summary and results.

References	Summary / Results
[34]	• Significant main effects of the siRNA encapsulation efficiency and particle size:<br>- PLGA concentration – positive effect<br>- volume ratio – positive effect<br>- interaction effect between the PLGA and the Ac-BSA concentration<br>• Optimal parameters for low siRNA load (770 ± 33 ng/mg nanoparticles):<br>- encapsulation efficiencies: 64.35 ± 2.78%<br>- average particle sizes: 265.0 ± 3.0 nm<br>• Optimal parameters for high siRNA load (2192 ± 115 ng/mg nanoparticles):<br>- encapsulation efficiencies: 70.63 ± 5.75%<br>- average particle sizes: 265.2 ± 1.8 nm<br>• Other measured parameters :<br>- the zeta potential of the particles were all negative, in the range: - 45.5 mV to - 37.5 mV<br>- release kinetics of Cy3-labelled siRNA from the PLGA optimal nanoparticles in TE buffer<br>- ToF-SIMS spectra to evaluate whether siRNA localized at the surface of the nanoparticles<br>- AFM topography images<br>- the ability of the PLGA matrix to protect the siRNA from nuclease activity by incubation of siRNA-loaded nanoparticles with Ribonuclease I ''A'' – protection up to 120h<br>- confirmation of biological activity of the siRNA in nanoparticles by extraction from the nanoparticles of siRNA directed against the mRNA encoding EGFP and used in gene silencing experiments in H1299 cells stably transfected with EGFP - the extracted siRNA was as efficient as the positive control siRNA in silencing the EGFP expression
[35]	• Seven new compounds with comparable potency were discovered with silenced factor VII by > 90% in vivo at the tested dose of 1.0 mg/kg<br>• Lipoplexes (simple ionic complexes with the nucleic acids) correctly predicted only two of 23 in vivo hit compounds (9%) while falsely rejecting the other 21 (91%) (hit compounds - reducing gene expression by more than 50% in the in vitro assay)<br>• The in vitro assay using LNPs (prepared by microfluidic method) identified 83% of the hit compounds<br>• The LNP-based in vitro assay identified all of the compounds with > 90% gene silencing in vivo as hit compounds, while the lipoplex-based assay falsely rejected all of them<br>• The four "false negatives" of the LNP-based in vitro assay are also interesting, because they may be indicative of liver-specific delivery<br>• The activity of the lipoplex is disproportionally dominated by the length of the hydrocarbon chain in the cationic lipid, only compounds with longer hydrocarbon chains (C12 and C14) were represented in the in vitro hits<br>• LNP hits included hydrocarbon chains of all tested lengths<br>• siRNA delivery efficacies in vitro and in vivo for different amine headgroups is a function of the hydrocarbon chain length<br>• The potency of the lipoplex increased with increasing hydrocarbon chain length, while the in vitro potency of the LNPs did not change significantly<br>• The in vivo potency of LNPs decreased with increasing hydrophobicity of the cationic lipids
[36]	• TT3 LLNs showed significantly higher expression of the firefly luciferase compared to other TT LLNs at a dose of 1.2 μg/mL of luciferase mRNA<br>• TT2-TT8 LLNs showed minimal to moderate inhibitory effects on Hep3B cells<br>• Significant positive correlation between transfection efficiency and entrapment efficiency<br>• No significant correlation between transfection efficiency and particle size, surface charge, and cell viability<br>• Increased TT3 and DMG-PEG2000 reduced mRNA delivery efficiency<br>• Increased DOPE facilitated mRNA delivery efficiency<br>• The optimal ratio for cholesterol ranged from 20 to 40<br>• Optimal formulation TT3/DOPE/Chol/DMG-PEG2000: 20/30/40/0<br>• Optimal formulation increased delivery efficiency over 20-fold compared to the best formulation (TT3 LLNs 1-13) identified in the first round of orthogonal optimization and over 350-fold compared to the original start-point TT3-DSPC LLNs<br>• Optimal formulations were over 65-fold more efficient than C12-200-DSPC LLNs<br>• Zeta potential showed a significant correlation with transfection efficiency<br>• The particle size of formulation LLNs with a low ratio of DMG-PEG2000, TT3/DOPE/Chol/DMG-PEG2000: 20/30/40/0 increased dramatically 5 h after, formulation TT3/DOPE/Chol/DMG-PEG2000: 20/30/40/0.75 were stable for a minimum of 2 weeks<br>• PEGylation improved particle stability and reduced particle size but hindered delivery efficiency

	• Optimal formulation of LLNs efficiently delivered hFIX mRNA in both wild-type and FIX-knockout mice and fully recovered the level of hFIX (791 mIU/mL at 1.1 mg/kg) to normal physiological values (500−1500 mIU/mL) in FIX-knockout mice.
[37]	• Decreased LNP size with increasing PEG composition<br>• Decreased encapsulation efficiency with increasing C12-200:mRNA weight ratio is caused by better complexation of more positively-charged ionized C12-200 lipid with negatively-charged mRNA<br>• Statistically significant trend of increasing serum EPO concentration with increasing C12- 200:mRNA weight ratio and with DOPE phospholipid, independent of the other formulation parameters.<br>• Statistically significant second-order effect between DOPE and increasing weight ratio C12- 200:mRNA<br>• Formulations with DOPE as the phospholipid resulted in significantly higher EPO production than formulations with DSPC<br>• The presence of DOPE in the formulation was the single strongest predictor of in vivo efficacy<br>• Independent of other varying formulation parameters, formulations with DSPC entrapped mRNA on average significantly better than DOPE<br>• The optimized formulation parameters: 10:1 C12-200:mRNA weight ratio, 35% C12-200, 16% DOPE, 46.5% cholesterol, and 2.5% C14-PEG2000 molar.<br>• The average efficacy with 15 μg total EPO mRNA injection in vivo: 7065 ± 513 ng/μL<br>• Particle size: 102 nm<br>• PDI: 0.158<br>• Encapsulation Efficiency: 43%<br>• pKa: 6.96<br>• Zeta Potential: -5.0 mV<br>• Luciferase protein generated by C-35 LNPs was expressed predominately in the liver (statistically significant), an approximately 3-fold increase in luciferase expression as measured by liver luminescence compared to the original formulation
[15]	• For a constant siRNA:lipidoid ratio:<br>- significantly higher (p-value < 0.001) loading of siRNA, proportional to the lipidoid content<br>- approx. five-fold increase in the loading when the lipidoid content was increased from 5% to 20%(w/w)<br>- no statistically significant differences (p-value > 0.05) in the particle size, PDI and encapsulation efficiency<br>• For fixed lipidoid content of 15% (w/w):<br>- significantly higher siRNA loading with increasing siRNA: lipidoid ratio<br>- siRNA loading was increased from 3.9 μg/mg with siRNA: lipidoid ratio of 1:30 (w/w), to as high as 10.6 μg/mg for a ratio of 1:10 (w/w)<br>- no significant differences (p-value > 0.05) in encapsulation efficiency and zeta potential<br>- significant increase in particle size and PDI for siRNA: lipidoid ratio of 1:10 (w/w) (p-value < 0.001) as compared to ratios of 1:15 and 1:20 (w/w)<br>• The inclusion of lipidoids did not interfere with the nanoparticle formation process, and statistically insignificant differences (p-value > 0.05) in the average hydrodynamic diameter (z-average) and PDI were observed when compared to non-modified PLGA nanoparticles<br>• Significantly higher zeta potential for the lipidoid-modified LPNs as compared to the non-modified PLGA nanoparticles<br>• Significant increase in encapsulation efficiency, from 3% in the case of PLGA NPs to >60% for all types of lipidoid-modified LPNs<br>• At 10% (w/w) lipidoid content, no statistically significant difference in encapsulation efficiency for L4-modified LPNs, L6-modified LPNs and Lmix-modified LPNs compared to L5-modified LPNs<br>• Encapsulation efficiency was significantly lower (p-value < 0.05) in the case of L6-modified LPNs as compared to that of Lmix-modified LPNs<br>• The type of PLGA core affects the encapsulation of siRNA in LPNs<br>• Significantly higher encapsulation efficiency (74.6%) was measured for LPNs prepared with high-molecular-weight PLGA (20 kDa) as compared to that of LPNs prepared using low-molecular-weight PLGA (10 kDa, 32.5%) at a constant lactic/glycolic acid molar ratio (75/25)<br>• No significant differences (p-value > 0.05) in particle size, PDI and zeta potential for PLGAs of a 20kDa molecular weight<br>• A two-fold increase in encapsulation efficiency for LPNs prepared with PLGA glycolic acid content higher than 25 mol% as compared to LPNs prepared with PLGA with a glycolic acid content below 15%<br>• Zeta potential in the latter case was markedly higher (+27.9 mV) as compared to that of the high-glycolic acid content formulation, which displayed an almost neutral zeta potential<br>• Significantly higher zeta potential and lower encapsulation efficiency for LPNs prepared with PLGA with different end group modifications (acid or amine), in contrast to LPNs with PLGA having esters as end groups<br>• Particle size and PDI were marginally affected by the lipidoid content and the siRNA: lipidoid weight ratio<br>• Slight increase in the particle size and PDI at higher siRNA: lipidoid ratios, i.e. 1:12.5 to 1:10, and at a lipidoid content of 13-18% (w/w). This could be attributed to an influence on the particle formation process due to the higher siRNA concentrations and the concurrent reduced amount of lipidoid<br>• Particle sizes for all formulations were within the range of 200 nm to 260 nm<br>• Initial experiments with L5-modified LPNs revealed that the ratio of siRNA: lipidoid, and not the lipidoid content, is a determining factor for the particle size and PDI<br>• Lipidoid content and the siRNA: lipidoid ratio contribute equally to reaching the overall CQAs of the formulation

	• Particle size was kept as one of the QTPP parameters, as it is widely known to influence pharmacokinetics, tissue distribution, tissue extravasation, uptake and/or accumulation within the clearance organs<br>• LPNs for pulmonary delivery, the upper limit of particle size was set to be <250 nm, because nanoparticles below this size evade the mucociliary and macrophage clearances thereby resulting in longer residence time when administered via the pulmonary route of administration<br>• Monodisperse particle size distribution is desirable for homogenous predictable performance and hence the QTPP for the PDI was set to <0.3<br>• Zeta potential increased proportionally as a function of lipidoid content and siRNA: lipidoid ratio but remained largely in the range of +5 to +15 mV<br>• The QTPP for zeta potential was set to >0 mV because nanoparticles with positive zeta potential have improved interactions with the plasma membrane. Excess surface charge is also associated with reduced colloidal stability, non-specific tissue internalization and shorter blood circulation time<br>• Zeta potential was highly dependent on the lipidoid content within the LPNs and increased from -20.9 mV at 5% (w/w) to +20.0 mV at 20% (w/w) at a lower ratio of siRNA: lipidoid (1:30)<br>• The effect of the ratio of siRNA: lipidoid on the zeta potential of the LPNs was only marginal and it remained on the positive side ranging up to +10 mV<br>• Lipidoid-modified LPNs show highly promising prospects for efficient and safe intracellular delivery of siRNA
[16]	• Critical formulation variables: L5 content and L5:Luc-ASO ratio (w/w)<br>• The optimised Luc-ASO-loaded LPNs, defined from the OOS, displayed high loading and mediated splice correction at well-tolerated, lower doses as compared to those required for reference L5-based lipoplexes, L5-modified stable nucleic acid-lipid nanoparticles or LPNs modified with dioleoyltrimethylammonium propane (conventional cationic lipid)<br>• The optimal Luc-ASO-loaded LPNs represent a robust formulation that mediates efficient intracellular delivery of Luc-ASO<br>• Significant impact type of PLGA used for the formulation of L5-modified LPNs on the loading of Luc-ASO:<br>- Luc-ASO encapsulation efficiency was very low, a maximum: 12–17% was achieved when using the PLGA type 75/25 with a molecular weight in the range 20–45 kDa<br>- for both lower and higher lactide/glycolide ratios, the encapsulation efficiency was very low, and it remained below 10% for all PLGA types irrespective of the molecular weight<br>- increasing the molecular weight and the lactide/glycolide molar ratio resulted in a slight, but not significant, increase in z-average, but these two formulation parameters had no significant impact on the PDI<br>- the Luc-ASO-loaded PLGA nanoparticles displayed a negative zeta potential ranging between −14.1 and − 19.9 mV, comparable to that of unloaded PLGA nanoparticles (approximately −25 mV)<br>- the hydrodynamic size increased slightly for higher molecular weight polymers, and when these displayed an increased lactide/glycolide molar ratio<br>- the PDI remained almost constant, except when incorporating the PLGA type with the highest lactide content (90%) into the LPNs<br>- the zeta potential was negative for LPNs prepared from the PLGA types with a lower lactide content (<75%) at a given molecular weight (20 kDa)<br>- however, inclusion of L5 (5%, w/w) in the nanoparticles resulted in a pronounced increase in the surface charge of the LPNs prepared from the higher molecular weight PLGAs or the PLGA types with a higher lactide content, respectively, and it reached a maximum of +25 mV<br>- the Luc-ASO encapsulation efficiency was increased (to a varying degree) for most of the LPNs as compared to their PLGA nanoparticle counterparts<br>- the most pronounced change was observed for the LPNs prepared with the PLGA type 90/10 (10 kDa), for which an increase from 1.4% (for PLGA nanoparticles) to 76.3%(for LPNs) was found. However, a bimodal particle size distribution was also observed for this formulation<br>- for the LPNs prepared with PLGA containing 75% lactide both particle size and zeta potential were significantly increased when the molecular weight of PLGA was increased from 20 kDa to 25–35 kDa, whereas the encapsulation efficiency remained constant<br>- the encapsulation efficiency for LPNs prepared with higher molecular weight PLGA (i.e., 25–35 kDa) decreased from approximately 100% to 25%, whereas there was a slight increase in the encapsulation efficiency for LPNs prepared with relatively lower molecular weight PLGA (i.e., 20 kDa)<br>- the particle size was decreased remarkably from~240 nm to ~195 nm when the LPNs were prepared with higher molecular weight PLGA (25–35 kDa)<br>- no significant difference in particle size for LPNs prepared with a relatively lower molecular weight PLGA (20 kDa).<br>- the PLGA type with a 75% lactide content and a molecular weight of 20 kDa was selected for further studies<br>• Effect of L5 on the physicochemical properties of LPNs:<br>- the average hydrodynamic diameter and PDI remained almost constant for Luc-ASO-loaded LPNs prepared with an L5 content between 5% and 20% (w/w)<br>- the zeta potential increased as a function of the L5 content from Luc-ASO encapsulation efficiency was generally very low and an approximately −15mVto +34mV<br>- no statistically significant difference (p-value> 0.5) in the Luc-ASO encapsulation efficiency was found when increasing the L5 content from 5% to 15%, however, an above three-fold increase in the Luc-ASO loading was achieved<br>- with an increase in L5 content from 15% to 20%, a pronounced decrease in encapsulation efficiency from 82% to 23% was observed<br>- this was accompanied by a decrease in loading from 6.1 to 2.3 μg Luc-ASO/mg LPNs.<br>- an L5 content of 15% (w/w) was chosen for further optimisation studies<br>• The L5:Luc-ASO Ratio Affects the Physicochemical Properties of LPNs:<br>- wide range of L5:ASO ratios (35:1 to 5:1, w/w) were tested to assess the effect on the physicochemical properties of the LPNs at a constant L5 content (15%, w/w).<br>- there was a slight, but not statistically significant, increase in the particle size up to a 7.5:1 ratio (225 nm to 247 nm).<br>- however, at a ratio of 5:1 (w/w), both the size and the PDI increased abruptly to 509 nm and 0.71, respectively.

	- the zeta potential was highest (+33.6mV) at the higher ratios of L5:ASO and lowest (−8.62mV) for the lowest ratio.<br>- a pronounced increase in the Luc-ASO loading was observed as a function of the L5:Luc-ASO ratio.<br>- the loading capacity was increased more than sixfold from approximately 2.33 to 14.17 µg Luc-ASO/mg LPNs, although the encapsulation efficiency decreased at lower ratios.<br>- the encapsulation efficiency remained almost constant in the range from 35:1 to 15:1 (~80%), but decreased at lower ratios.<br>• Statistical Optimisation of L5-Modified LPNs and Contour Profiling of the Response Variables:<br>- the z-average was not significantly affected by the independent variables and ranged within 179–205 nm<br>- similar results were also observed for the PDI, which ranged from 0.056 to 0.164. A minor increase in the PDI of approximately 0.164 was found at higher lipidoid content and L5:Luc-ASO ratio, which was still within the acceptable ranges of the QTPP<br>- the zeta potential of the LPNs increased with a higher L5 content at all L5:Luc-ASO ratios. The influence of the L5:Luc-ASO ratio on the zeta potential of the LPNs was only evident at a lower L5 content. With a higher L5:Luc-ASO ratio, a concomitant increase in the zeta potential was found. Notably, for all formulations, the zeta potential remained positive and was within the range of +3 mV to +37 mV<br>- the encapsulation efficiency was highly dependent on both the L5 content and the L5:Luc-ASO ratio. A clear declining trend in encapsulation efficiency was noted with an increase in L5 content beyond 16% (w/w), irrespective of the L5:Luc-ASO ratio<br>- at lower L5 content (<15%), very high encapsulation efficiencies (>90%) were obtained at higher L5:Luc-ASO ratios. At higher L5 content, the encapsulation efficiency decreased proportionately with the increase in L5 content. The contour plot for the Luc-ASO loading revealed a non-linear correlation with an optimal range of 14–18% L5 content, within which the loading varied from 6 to 12 µg Luc-ASO/mg LPNs. In contrast to the declining encapsulation efficiency observed in this region when decreasing the L5:Luc-ASO ratio, the Luc-ASO loading displayed a pronounced increase due to the higher theoretical loading<br>- of the biological responses, i.e., in vitro splice correction and effect on cellular viability were also determined. Each formulation from the experimental design was tested at five different Luc-ASO concentrations. The obtained splice correction at each Luc-ASO concentration was normalised to the positive control and plotted as a function of the Luc-ASO concentration, followed by fitting the data to a linear function by using regression analysis. The slope of the linear fit represents the splice-correction rate<br>- as observed in the OFAT experiments, the ability to mediate splice correction was highly dose-dependent. The luciferase activity resulting from the splice-correction event was generally highest at 154 nM Luc-ASO, except for the LPNs with the highest L5 content, which mediated efficient splice correction even at 10-fold lower concentrations<br>- the splice-correction rate increased with higher L5 content<br>- the extent of enhancement was more pronounced at higher L5:Luc-ASO ratio, as compared to that determined at lower ratios<br>- a similar trend was found for all formulations concerning their effects on cell viability. The formulations were better tolerated when the L5 content was lower and the L5:Luc-ASO ratio was higher, as compared to that of LPNs with a higher L5 content<br>- for instance, LPNs prepared with an L5 content of 20% (w/w) and an L5:Luc-ASO ratio of 30:1 had an IC50 of 30 nM, while the formulation prepared with 10% (w/w) and an L5:Luc-ASO ratio of 10:1 displayed a remarkably high IC50 value of ~398 nM<br>- however, for most formulations, the IC50 value was within the range 120–260 nM<br>• The OOS area:<br>- 14–17% (w/w) L5 content and L5:Luc-ASO ratios from 11:1 to 21:1<br>- the extent of the OOS was mainly defined by the biological responses, and it was restricted by the splice correction efficiency and the effect on cell viability<br>- in addition, the zeta potential was also an important factor in determining the extent of the OOS<br>- finally, five formulations within the OOS were selected for preparation to assess the validity of the model. These formulations all displayed CQAs according to the pre-set QTPP, and hence the constructed design space was found to be valid
[38]	• Lipid type and lipid concentration were the only significant factors<br>• Cephalin lipid identity had the strongest effect, with $p = 0.0018$<br>• Cephalin LNPs with a ratio of total lipids to RNA of 18:1 (w/w), low lipid concentration, and medium particle concentration yielded a 7-fold increase in luciferase expression over the original formulation<br>• Flow cytometry revealed that all of the formulations enhanced the eGFP expression in human skin cells and paralleled the enhanced delivery with cephalin, DDA, and DOTAP LNPs observed with luciferase imaging studies<br>• Epithelial cells and fibroblasts were found to comprise the majority of the resident skin cell population and the immune cells were found to express more of the administered RNA, concerning their proportion of the total cell population.
[39]	• Particle size and PEG to phospholipid ratio (PEG/PL) are other key factors for liver-specific gene expression in addition to the examined formulation factors<br>• The optimized formulation showed better gene expression compared to other lipid formulations from industry leaders<br>• DoE with multiple responses has clear advantages over conventional DoE with only one response, and it is capable of efficiently revealing the parameters that are important in predicting optimal formulations<br>• 1st DoE: identification of critical factors for mRNA-loaded LNPs by DSD:

	- particle diameters: from 26 to 107 nm<br>- PdI: from 0.126 to 0.439<br>- gene expression in the tested tissues: from 104 to 108 RLU/mg protein<br>- one formulation showed a much lower mRNA encapsulation efficiency (42.4%) and a larger diameter (107 nm) compared to the others (over 90% and less than 70 nm, respectively), and was, therefore, omitted from the subsequent correlations<br>- Nluc expression between the two organs – no significant correlation (R2=0.392, p=0.087)<br>- Nluc expression in the liver against LNP diameter - significant positive correlation (R2=0.747, p=0.0031)<br>- Nluc expression in the liver against and PdI – significant negative correlation (R2=0.670, p=0.00094)<br>- uniform particles with diameters of at least 40 nm are required for gene expression in liver<br>- gene expression in the spleen against diameter - significant positive correlation (R2=0.422, p=0.024)<br>- gene expression in the spleen against PdI - significant negative correlation (R2=0.169, p=0.182)<br>- relatively larger diameter is required for gene expression in the spleen<br>- three statistically significant main factors (CL, PL, and %PEG) were found concerning the increase in LNP diameter<br>- two statistically significant main factors (CL and PL) and two secondary factors (CL*%PEG and PL*%PEG) were identified concerning the decrease in PdI<br>- three statistically significant main factors (CL, PL, and %CL) and two secondary factors (CL*PL and CL*%CL) were found concerning the expression of Nluc in the liver<br>- three statistically significant main factors (CL, %CL, and %PEG) and one secondary factor (%CL*%PEG) were found concerning Nluc expression in the spleen<br>- CL4H6 and DSPC can be used to synthesize significantly large, uniform and potent LNPs<br>- higher %CL was important in terms of maximizing gene expression<br>- the mRNA/lipid ratio was not a significant factor for any of the responses<br>- for both %PL and %PEG, a clear conclusion was not obtained by the above model fitting<br>- it should be noted that the top two formulations in the liver showed significantly different gene expressions in off-target organs and that the major difference in the formulation parameter between the two LNPs was the PEG/PL ratio<br>- specifically, formulation with a higher PEG/PL ratio of 0.5 showed a higher liver-specificity (3 to 9-fold) compared to the LNPs with a lower ratio of 0.06<br>- a correlation between the PEG/PL ratio and liver specificity was observed in the library. These results suggest that the LNPs with higher PEG/PL ratios are rapidly eliminated from the blood circulation and accumulate in the liver whereas those with lower PEG/PL ratios circulate in the blood longer and are distributed in the liver and off-target organs as well<br>• 2nd DoE: identification of optimal mRNA-loaded LNPs by L18-Taguchi FFD<br>- the LNP diameter, PdI, and Nluc expression in the liver in the case of library B were significantly higher, lower, and higher, respectively, compared with those of library A<br>- Nluc expression in the liver and spleen – no significant correlation (R2=0.146, p=0.130)<br>- particle diameter and gene expression in the liver - no significant correlation (R2=0.0454, p=0.411)<br>- diameter and gene expression in the spleen - a significant positive correlation (R2=0.301, p=0.023)<br>- optimal range of particle diameter is different between the liver and spleen<br>- liver-specific formulations of particles showed significantly lower diameters and higher PEG/PL ratios<br>- two statistically significant main factors (%CL and %PEG) to the increase in LNP diameter<br>- no statistically significant factor to the decrease of PdI<br>- two statistically significant main factors (%PL and %PEG) to the increased Nluc expression in the liver<br>- one statistically significant main factor (%PEG) to an increased Nluc expression in the spleen<br>- LNPs (B13), composed of CL4H6/ESM/chol/PEG-DMG at a molar ratio of 60/5/35/1.5, best met the criteria for both Nluc expression in the liver and liver-specificity. TEM analyses revealed that the this-LNPs formulation had a spherical structure<br>• Characterization of the optimized mRNA-loaded LNPs:<br>- the highest serum EPO concentration was observed for the B-13-LNPs over the other two LNPs, a result that was similar to that for Nluc expression<br>- the hEPO level of the B-13-LNPs (8756 ng/mL) was 1.24-fold higher than that of the C12-200-LNP which was optimized for hEPO expression at a 1.5-fold higher dose (0.75 mg mRNA/kg). The C12-200-LNPs showed an expression of hEPO at therapeutically relevant levels in non-human primates and restored blood clotting time in haemophilia B model mice by the expression of human factor IX at the same dose. These collective findings suggest that the B-13-LNPs induce therapeutically relevant levels of protein expression<br>- distribution in non-liver organs (spleen, lung, and kidneys) and blood in ApoEdeficient mice was significantly higher than those in wild-type mice, whereas that in the liver was similar<br>- the B-13-LNPs were localized in hepatocytes (extravascular region, arrows) in wild-type mice but not in ApoE-deficient mice. Nluc expression in livers in ApoE-deficient mice was significantly (approximately 5-fold) lower than that in wild-type mice whereas that in the spleen was not significant. These collective results suggest that ApoE facilitated the rapid clearance by hepatocytes, liver-specific distribution and gene expression of the B-13-LNPs<br>- compared with other lipid formulations from industry leaders like MC3-LNPs, the B-13-LNPs showed approximately a 2.5-fold higher Nluc expression in the liver

	- the mCherry-encoding mRNA-loaded B-13-LNPs also showed a higher expression in hepatocytes compared with the MC3-LNPs<br>- biosafety was confirmed of the optimized mRNA-loaded LNPs<br>- B-13-LNPs have better properties for safe and effective treatment compared with the recently developed formulations<br>• Main conclusions:<br>- the desired size ranges for efficient gene expression in the liver and liver-specific gene expression and the results revealed that the PEG/PL ratio can be an indicator of liver-specificity<br>- DSPC was significantly preferable to DOPE for gene expression in the liver but not in the spleen<br>- DOPE-containing LNPs showed significantly lower gene expression in the liver compared to DSPC-containing LNPs<br>- although the precise reason why optimal PL is different between pH-sensitive cationic lipids and lipidoids could be related to the mechanism of uptake in hepatocytes<br>- approximately 60 nm would be a critically minimal diameter and a diameter from 60 to 100 nm would be the optimal range to maximize total gene expression in the body (e.g. for secretory protein replacement therapy)<br>- diameter from 60 to 70 nm would be optimal from the viewpoint of liver specificity, which would be important for non-secretory protein replacement therapy and genome editing<br>- chemical structure and the molar ratio of lipids were significant regulatory factors for both formulation diameter and gene expression<br>- formulation diameter also significantly affected gene expression<br>- formulation diameter is a confounding factor<br>- higher PEG/PL ratios facilitate rapid blood clearance and hepatic accumulation of the LNPs<br>- the diameter and PEG-lipid to-phospholipid ratio were found to be significant parameters for liver-specific expression, which were newly found in the present study.<br>- the optimized B-13-LNPs showed an ApoE-dependent liver-specific biodistribution and gene expression and exhibited significantly higher gene expression efficiency compared to other lipid formulations from industry leaders
[17]	• Significant differences were not observed in the z-average and PDI, and the values were within the range of the QTPP<br>• A ~1.9-fold increase in the loading was observed when the L5 content was increased from 15 to 25% at L5:TNF-a siRNA ratios from7.5:1 to 15.0:1<br>• The loading dropped to 1.5-fold when the ratio was 5.0:1<br>• The zeta potential increased with the L5:TNF-a siRNA ratio due to an increase in the content of cationic L5<br>• Z-average and PDI of the LPNs remained almost constant at all tested L5:TNF-a siRNA ratios<br>• Significant decrease in the zeta potential was observed as the L5:TNF-a siRNA ratio was decreased (corresponding to a higher amount of siRNA without increasing the L5 content)<br>• The L5 content and the L5:TNF-a siRNA ratio influence the zeta potential and the siRNA loading<br>• The encapsulation efficiency was neither affected by the L5 content nor the L5:TNF-a siRNA ratio, and no significant differences were observed the responses were within the QTPP (>60%)<br>• The encapsulation efficiency increased (from 64.4 to ~80%) as the L5 content increased from 15 to 25%<br>• The relatively lower encapsulation efficiency could be due to the high pH (7.5) during the emulsification process as L5 might not be fully protonated<br>• Hence, a value higher than 60% was set for the encapsulation efficiency, while a higher siRNA loading (> 6 μg siRNA/mg LPNs) would have a positive impact on the gene silencing effect and reduction in cytotoxicity<br>• Both the L5 content and the L5:TNF-a siRNA ratio affected the gene silencing<br>• At an siRNA concentration of 5.6 nM for LPNs having 15% L5 content, the TNF-a mRNA expression was ~60%, and the expression was at least 80% with the remaining LPNs.<br>• At the highest tested siRNA concentration (55.8 nM), the TNF-a mRNA expression generally decreased as the L5 content increased at a constant L5:TNF-a siRNA ratio.<br>• LPNs containing an L5 content of 15, 20, and 25% with an L5:TNF-a siRNA ratio of 15:1 were most efficient in causing a significant concentration-dependent decrease in TNF-a mRNA expression.<br>• Of all the LPNs tested for cell viability, only a few of them (15%, 10.0:1 and 7.5:1; 20%, 7.5:1, and 5.0:1; 25% 15.0:1) seemed to have cytotoxic potential but none of them caused > 50% loss in cell viability even at 222.8 nM.<br>• In terms of cytotoxicity and TNF-a gene silencing effect, formulations having an L5 content and L5:TNF-a siRNA wt. the ratio of 15%, 15.0:1 and 20%, 15.0:1 exhibited a balanced profile and were consequently used for model validation<br>• The differences in z-average were not significant in the design space<br>• The z-average decreased considerably when the L5 content was higher than 20% and the L5:TNF-a siRNA ratio was >7.5:1, and vice versa<br>• There was a gradual increase in the PDI (from ~0.100 to 0.175) as the L5 content increased from 15 to 25%. A minor increase in the PDI was observed when the L5:TNF-a siRNA ratio decreased at an L5 content of 15%<br>• The zeta potential increased gradually from 6.7mV to a maximum of 39.0mV<br>• The encapsulation efficiency remained almost constant (64–78%) throughout the design space, which may indicate that the encapsulation efficiency is more dependent on the process of preparing the LPNs rather than on the independent variables.<br>• The siRNA loading was affected by both independent variables L5 content and L5:TNF-a siRNA ratio<br>• When keeping the L5 content constant and varying the L5:TNF-a siRNA ratio, it was observed that the siRNA loading decreased 3-fold when the L5:TNF-a siRNA ratio was increased from 5.0:1 to 15.0:1.

	• The L5:TNF-a siRNA ratio displayed a greater impact on the loading, which was expected as a higher amount of siRNA would correspond to increased loading per weight of LPNs<br>• To test the performance of the formulations loaded with TNF-a siRNA in a biologically relevant system, the IC50 values were calculated as a function of the TNF-a siRNA concentration in formulations responsible for half-maximal inhibition of TNF- a mRNA expression in macrophages<br>• The IC50 values for transfection efficiency of all formulations ranged from 10.2 to 50.0 nM<br>• Formulations displaying the highest L5:TNF-a siRNA wt. ratio, i.e., the least amount of TNF-a siRNA relative to L5, at 15, 20, and 25% L5 had transfection efficiencies below 20 nM<br>• The IC50 value for naked TNF-a siRNA was found to be 63.7 nM<br>• Fold-change in TNF-a mRNA inhibition relative to naked siRNA was obtained by the ratio of IC50 values of TNF-a siRNA-loaded LPNs and naked TNF-a siRNA<br>• At a constant L5 content, the fold-change increased as per the quadratic model fitting from 7.5:1 to 15.0:1 ratio.<br>• When the L5:TNF-a ratio was kept constant, the fold change generally increased with an increase in the L5 content. Interestingly, LPNs with an L5 content of 15 and 25% had similar IC50 values<br>• The dose-response of siRNA LPNs demonstrated a lack of cytotoxic potential for different formulations tested at certain concentrations for gene silencing and only a few of them (15%, 10.0:1 and 7.5:1; 20%, 7.5:1; 25% 15.0:1) seemed to have cytotoxic potential. The possibility of potential apoptotic and necrotic effects of the particles should not be disregarded since it may not be apparent from the MTT assay. However, at TNF-a siRNA concentrations of 100 and 200 nM, no significant apoptotic or necrotic cells were observed for 15%, 15.0:1 LPNs<br>• The toxicity of the formulations is most likely caused by the L5 component<br>• The concentration of TNF-a siRNA in the most optimal formulations required to knock down between 60 and 69% of TNF-a was found to be 27.8 nM, while a concentration of 55.8 nM was required for >80% knockdown. At these concentrations, none of the most optimal formulations displayed any cytotoxic potential.<br>• We found a linear increase in the TNF-a gene silencing effect when the L5:TNF-a siRNA ratio was increased from 7.5:1 to 15.0:1 at 15, 20, and 25% L5 content. LPNs with a 5.0:1 ratio silenced TNF-a gene expression more efficiently than 7.5:1.<br>• the highest tested concentration of siRNA, the gene expression of TNF-a in LPS-activated macrophages treated with LPNs containing 15, 20, and 25% L5 and having an L5:TNF-a siRNA ratio of 15.0:1 was only 19, 12, and 6%, respectively.<br>• LPNs composed of 15, 20, and 25% L5 having an L5: TNF-a siRNA ratio of 15.0:1 were found to be most optimal for gene silencing in macrophages, as determined from their IC50 values<br>• Optimal Operating Space: 15%, 13.5:1 and 25%, 15.0:1
[18]	• The introduction of Chol-PEG400-SP, Chol-PEG400-Man, and Chol-PEG2000-WRK improved Luc mRNA expression in the liver<br>• SMWLLNs/ Luc containing three derivatives achieved the highest bioluminescence intensity compared with other groups<br>• The formulation of SMW-LLNs was chosen for further optimization<br>• No significant difference in the size, zeta potential, and encapsulation efficiency among formulations used for optimization, with different derivatives of cholesterol.<br>• All three input variables showed a good fit to the second-order polynomial model<br>• Increasing the molar ratio of Chol-PEG400-SP, Chol-PEG400-Man, and Chol-PEG2000-WRK resulted in greater bioluminescence intensity of the liver within a certain range.<br>• The optimal ratio of Chol-PEG400-SP, Chol-PEG400-Man, and Chol-PEG2000-WRK in optimal LLN (O-LLNs) were forecasted to be 4.37%, 6.29%, and 3.38%, respectively<br>• Bioluminescence intensity in the liver using O-LLNs was greater than the Origin-LLNs 6 h after i.v. injection of Luc mRNA<br>• The optimized LLNs (O-LLNs) by CCD succeeded in the functional delivery of Cre-recombinase (Cre) mRNA, human erythropoietin (hEPO), and mCherry mRNA, respectively<br>• The O-LLN/mRNA complex showed a spherical morphology in the transmission electron microscopy (TEM) image and had a mean particle size of 136 nm (DLS)<br>• The surface charge of O-LLNs/mRNA complex was positively charged (zeta potential = 35 mV)<br>• mRNA/LLNs could be physically stable at 4 °C for at least 2 weeks<br>• O-LLNs/mRNA with various mRNA concentrations showed negligible cytotoxicity after 24 h (>80% cell viability), indicating an excellent cell compatibility of O-LLNs/mRNA<br>• O-LLNs/Cy5 significantly inhibited macrophages uptake compared with Chol-PEG400-SP-free O-LLNs/Cy5<br>• O-LLNs were one of the potential carriers for in vivo liver-targeted mRNA delivery<br>• O-LLNs/hEPO induced the strongest expression of hEPO in the blood of C57BL/6 mice with intravenous injection of the preparations consisting of hEPO mRNA, compared to Origin/hEPO ($p < 0.001$) and MC3/hEPO ($p < 0.05$).<br>• mCherry highly expressed in the albumin antibody-labeled hepatocytes.<br>• O-LLNs optimized by CCD were one of the highly efficient liver-targeted systems for mRNA delivery according to the investigation of the distribution and functional delivery.<br>• O-LLNs achieved more than 8% of base editing efficiency, which was 2.36-fold of the Origin-LLNs for Pah^{enu2} mice treated by Origin-LLNs or O-LLNs containing CBE mRNA and sgRNA at a total dose of 1 mg kg–1 (mRNA/sgRNA, 1/1, wt/wt).<br>• Optimized lipid-like nanoparticle with 4.37% Chol-PEG400-SP, 6.29% Chol-PEG400-Man, and 3.38% Chol-PEG2000-WRK, which can synergistically prolong systemic circulation, as well as increase liver targeting and hepatocyte uptake<br>• O-LLNs resulted in functional mRNA delivery to the livers in Ai9 mice and more than 8% base editing efficiency in PKU mice.
[19]	• The minimum of 2740 $ costs of T7RNAPmand NTPs expressed per g of RNA is at 1.5 × 10–8M T7RNAP concentration, at 40.8mM NTP concentration and at an RNA yield of 4.34 g/L,
[20]	• With high ASO loading, an increase in mixing speeds or cycles did not affect particle size and %EE<br>• Optimal condition: ethanol-to-buffer injection followed by 10 cycles of mixing at medium speed 0.5 mL/s, sufficient to produce homogeneous LNPs

	• PEGylated lipid was critical for LNP formation<br>• Increasing the PEGylated lipid content significantly (*P* < 0.0001) reduced the mean particle size, i.e., lipids containing 1.5, 3, and 5 mol% of DSPE-PEG2000 resulted in LNPs with average diameters of ~120, ~80, and ~60 nm, respectively<br>• DSPE-PEG2000 incorporated at 1.5 mol% of the total lipids produced unimodal nanoparticles with a mean diameter of ~120 nm whereas more PEG increased polydispersity<br>• 5 mol% of DSPE-PEG2000 even produced a subpopulation, possibly due to the formation of small DSPE-PEG2000 micelles<br>• %EE of ASO was mainly determined by the N/ P ratios. Percent ASO encapsulation was higher than 80% when the N/P ratio was higher than 1, i.e., there were excess complexation sites in MC3; whereas decreasing the N/P ratio to 0.5 significantly reduced %EE to ~50%<br>• Similar results were also found when MC3 was replaced by another cationic lipid DOTAP or ASO-1 was replaced by ASO-2 using the same formulation conditions such as pH and buffer<br>• ASO-loaded LNPs prepared by the HTS approach and microfluidic formulator showed a similar structure of a condensed core with multiple stacked lipid layers (cryo-TEM images)<br>• Compared with N/P = 1, both methods running at higher N/P ratios produced LNPs with more condensed cores without evident multilamellar structures<br>• The two LNP preparation methods showed similar results:<br>- LNP size decreased while polydispersity increased with increasing PEG content<br>- compared to PEG contents, LNP sizes were less affected by total lipid concentrations (up to 2 mM) and N/P ratios<br>- excess ASO loading (N/P ratio < 1) resulted in significant decrease in % EE.<br>- The HTS approach successfully predicted the dependence of particle size and polydispersity on the PEGylate lipid content, shown by strong correlations with linear regression R2 > 0.9.<br>• 1.5 mol% of DSPE-PEG2000 and the N/P ratio ≥ 1 would produce optimal LNP formulations with a homogeneous and stable particle size as well as high ASO loading<br>• HTS approach decreased material consumption by ~10 folds and improved processing outputs by ~100 folds, compared to the larger scale microfluidic preparation
[40]	• siRNA encapsulation values showed approximately 90% in all LNP-siRNA systems<br>• The R2 values for particle size and PDI of empty LNPs before dialysis were 0.94 and 0.99, respectively, these values after dialysis were 0.99 and 0.92, respectively.<br>• Similarly, the R2 values for particle size and PDI of siRNA-containing systems before dialysis were 0.96 and 0.95, respectively, and 0.91 and 0.95, respectively, after dialysis<br>• FRR and FRR × FRR showed a remarkable effect (P < 0.01) on the particle size and PDI of both empty LNPs and LNP-siRNA systems<br>• LNP-siRNA systems, the lipid concentration showed a significant effect on the particle size and PDI<br>• A trend of increase in particle size following dialysis was observed with both empty LNPs and LNP-siRNA systems<br>• The particle size of LNP-siRNA systems was affected by the FRR and lipid concentration and confirmed that the particle size will likely increase if a low FRR (i.e., more aqueous) and a high lipid concentration are used<br>• Lipid concentration influenced (with statistical significance) the particle size of LNP-siRNA systems but not empty LNPs<br>• FRR is a key parameter to control the PDI of LNPs because the diffusion rate in microfluidics is reduced at a low FRR<br>• Increased FRR (to 3) was associated with a decreased PDI in both empty LNPs and LNP-siRNA systems. However, in the range of 3–5 FRR, no impact on PDI was observed<br>• Lipid concentration was found to have a greater impact on the PDI of LNP-siRNA systems than on that of empty LNPs, and there was a tendency for the PDI to increase with increasing lipid concentration<br>• Reduction in the PDI was observed following dialysis in LNP-siRNA systems<br>• LNP-siRNA systems showed substantial reductions in the PDI following dialysis, while the PDI of empty LNPs generally remained the same or slightly increased following dialysis<br>• The increase in the particle size that occurred after dialysis was much higher with LNP-siRNA systems than with empty LNPs<br>• Empty LNPs displayed a similar small particle size at FRR = 3 and 5; however, the particle sizes (and PDI) before and after dialysis were much larger at FRR = 1.<br>• For LNP-siRNA, the particle sizes stayed relatively consistent before dialysis regardless of FRR.<br>• A similar observation was made for the post-dialysis samples, all formulations displayed similar sizes that are generally larger than their pre-dialysis counterparts. This indicates that the presence of anionic cargo such as siRNA limits the rearrangement of lipids.<br>• The method of pH neutralization process in manufacturing is important to obtain LNP-siRNA with desirable properties (defined particle sizes, low polydispersity indices, and high entrapment)<br>• LNP-HA systems (LNP containing HA - complexes with HA with molecular weight similar to that of siRNA) demonstrated that the degree of change in particle size caused by FRR was smaller than that of empty LNPs but larger than that of LNP-siRNA systems, suggesting that the binding affinity of small vesicles with anionic cargos plays a significant role in the formulation of the LNP complexes. Further studies are required to determine how different types of anionic cargos (modified nucleic acids or peptides) influence nanoparticle formation.
[21]	• CD8 T cell response heavily depends on the LNP composition<br>• Critical effects:<br>- Single effects:<br>DMG-PEG2000: PEG-lipid,<br>DSG-PEG2000: PEG-lipid and ionizable lipid<br>DSPE-PEG1000: PEG-lipid and ionizable lipid<br>- Interaction effects:

	DMG-PEG2000: PEG-lipid and DOPE<br>DSG-PEG2000: PEG-lipid and DOPE; ionizable lipid and PEG-lipid; ionizable lipid and DOPE<br>DSPE-PEG1000: PEG-lipid and DOPE<br>- Quadratic effects:<br>DMG-PEG2000: PEG-lipid<br>DSG-PEG2000: PEG-lipid<br>DSPE-PEG1000: DOPE, ionizable lipid, PEG-lipid<br>• T cell response is most sensitive to changes in %PEG-lipid, followed by changes in %DOPE and %ionizable lipid respectively for all types of PEG lipid<br>• Changes in %ionizable lipid have a bigger mean impact on T cell response for DSG-PEG2000 LNPs (S= 27%) than for DMG-PEG-2000 (S=21) and DSPE-PEG1000 LNPs (S=16).<br>• The ratio of T cell response sensitivity to changes in %PEG-lipid over changes in %ionizable lipid is highest for DSPE-PEG1000LNPs (49/16 = 3), followed by DMG-PEG2000 (42/21 =2) and DSG-PEG2000 (37/27=1.4).<br>• quasi-constant sensitivity of the immune response to %DOPE (around 36%), only the ratio %PEG-lipid/%ionizable lipid depends on the PEG-lipid type.<br>• For all PEG-lipid types, the predicted optimal T cell response region corresponded to high percentages of the ionizable lipid SS-EC (>50%) and low percentages of PEG lipid (<0.6%) and DOPE (<10%)<br>• DSPE-PEG1000 LNPs were excluded - the model could not be validated and unpredictable behaviour and visual toxicity in mice (lethargy, pain symptoms) were observed<br>• Optimal LNPs composition:<br>DMG-PEG2000: ionisable lipid 56.5%, DOPE 5.25%, Cholesterol 37.75%, PEG-lipid 0.5%<br>DSG-PEG2000: ionisable lipid 64.4%, DOPE 8%, Cholesterol 27.1%, PEG-lipid 0.5%
[22]	• Exemplary optimal LNP formulation candidates:<br>- max potency (weight 1.0, predicted 100.3%), min size (weight 0.2, predicted 68.2 nm): 5% PEG, 48.07% Helper, 10% Ionsable lipid, 36.93% Cholesterol, Ionsable lipid type H102, flow rate 2.57 mL/min, desirability 0.9895<br>- max potency (weight 1.0, predicted 94.2%), min size (weight 0.2, predicted 78.8 nm), force H103, 5% PEG, 45.01% Helper, 10% Ionisable lipid, 39.99% Cholesterol, Ionisable lipid type H103, flow rate 2.56 mL/min, desirability 0.8485<br>- max potency (weight 1.0, predicted 101.5%): 5% PEG, 41.88% Helper, 10% Ionsable lipid, 43.12% Cholesterol, Ionsable lipid type H102, flow rate 1.00 mL/min, size (predicted) 100.6 nm, desirability 0.9954
[23]	• In vitro transcription space-time yield (STY) significant factor:<br>- volume flow rate - the biggest influence<br>- nucleotide and enzyme concentrations and their interactions with each other and with the volume flow rate<br>• Inline Diafiltration by SPT significant factor:<br>- TMP (transmembrane pressure) and the volumetric flow rate of the exchange buffer - the most significant effects on the buffer exchange BE and the volumetric concentration factor VCF<br>- optimum is at a TMP of 1.4 bar and a buffer exchange flow rate of 13 mL/min<br>• LNP significant factor:<br>- encapsulation efficiency (EE) – optimal > 80%<br>- mRNA concentration – optimal approx. 0.26–0.30 g/L<br>- volume flows of the aqueous (optimal approx. 250-295 mL/min) and organic phases<br>- lipids concentrations and their interaction – optimal PEG lipids concentration approx.1.2–1.6 g/L, cholesterol concentration approx. 4.7-6.3 g/L<br>- pH influence on EE
[24]	• MiRNAs are the most studied small ncRNAs<br>• More in vivo studies are required to determine whether miRNAs target specific cells under physiological conditions. Nevertheless, the current knowledge represents only a small fraction of the landscape of gene regulatory potential<br>• The most important characteristics of nanosystems are zeta potential, particle size and size distribution, shape, morphology, cellular uptake, and transfection efficiency<br>• The positively charged nanoparticles can encapsulate negatively charged genetic material by electrostatic interaction<br>• Cationic liposomes and cationic polymers generally represent safe and efficient carriers for successful gene delivery<br>• Implementing QbD concept could bring a lot of advantages essential to developing non-viral vectors with reproducible physicochemical properties suitable for large-scale production<br>• Cationic lipids for gene delivery have become a major research tool for transferring genetic material into cells and there is great potential for progress in this direction
[25]	• The specific combination of ionizable lipid and phospholipid in the LNP design yields high transfection efficiency *in vitro*<br>• high-performing LNPs have no toxicity to both the pregnant mice and fetuses<br>• LNPs composed of ionizable lipid, C12-200, with DOPE phospholipid, are required for potent mRNA delivery to trophoblasts<br>• type of ionizable lipid was a main effect for apparent pKa (p<0.001) with C12-200 in LNPs yielding lower apparent pKa values compared to DLin-MC3-DMA

	• LNP A10 (35% C12-200, 10% DOPE, 1.5% PEG, 53.5% Cholesterol; 130.2 nm HDD, 0,064 PDI, 56.5% EE, 6.607 pKa) yielded ~190,000-fold higher luciferase expression compared to phosphate-buffered saline (PBS)-treated cells (p<0.0001)<br>• LNPs A5 (25% C12-200, 22% DOPE, 2.5% PEG, 50.5% Cholesterol; 142.2 nm HDD, 0,126 PDI, 64.9% EE, 6.067 pKa), A8 (25% C12-200, 22% DOPE, 3.5% PEG, 49.5% Cholesterol; 131.2 nm HDD, 0.096 PDI, 64.0% EE, 6.031 pKa), and A14 (35% C12-200, 16% DOPE, 2.5% PEG, 46.5% Cholesterol; 110.2 nm HDD, 0.139 PDI, 64.0% EE, 5.619 pKa) had the next highest luciferase expression compared to PBS-treated cells (p<0.001)<br>• No LNPs prepared with DLin-MC3-DMA or DSPC yielded high luciferase expression<br>• The majority of LNP formulations are not toxic to BeWos<br>• Type of ionizable lipid (p=0.018) and type of phospholipid (p=0.017) were significant factors affecting transfection<br>• C12-200 or DOPE in LNPs yielded the strongest luciferase expression overall compared to the other LNP components<br>• Maximal transfection efficiency occurs when C12-200 and DOPE are both included in the LNP formulation (p=0.0105)<br>• The model found several pairwise interactions between the type of ionizable lipid and PEG amount (p=0.036), type of phospholipid and PEG amount (p=0.034), and type of ionizable lipid and type of phospholipid (p=0.0105)<br>• Type of ionizable lipid (p=0.005), type of phospholipid (p=0.018), ionizable lipid amount (p<0.0001) and phospholipid amount (p=0.016) were significant factors affecting the viability of BeWos<br>• The two LNPs that resulted in significantly lower viability compared to the controls were comprised of low amounts of DLin-MC3-DMA and DOPE – 25% and 10%, respectively<br>• The model found pairwise interactions between the type of phospholipid and phospholipid amount (p<0.0001) and type of phospholipid and ionizable lipid amount (p<0.0001)<br>• Results in vitro do not correlate with delivery efficiency in vivo, optimal LNP composition for high placenta delivery in vitro is different than optimal LNP composition for high in vivo mRNA delivery in trophoblasts<br>• Optimal LNP A10 (35% C12-200, 10% DOPE, 1.5% PEG, 53.5% Cholesterol; 130.2 nm HDD, 0,064 PDI, 56.5% EE, 6.607 pKa)<br>• LNP A10 is nontoxic to both the dams and the fetuses following treatment<br>• DSD method identifies LNP formulation capable of safely delivering mRNA to the placenta, providing an opportunity to treat placental dysfunction during pregnancy0
[26]	• HEPES-acetate concentration in buffer treatment is critical for stabilizing the size of nascent LNPs<br>• LNPs can be dialyzed against formulation buffer after stabilization without significant variation in sizes<br>• buffer strength and pH have critical impacts<br>• optimal LNPs diameter 60–180 nm (± 10 nm), PDI ≤ 0.200
[27]	• Desirable LNPs have the following physicochemical characteristics:<br>- particle size (Z-average diameter) between 80 and 100 nm,<br>- polydispersity index (PDI) less than 0.2<br>- encapsulation efficiency (EE) at least 80%,<br>- neutral zeta potential<br>• The presence of PEG was crucial for improved physicochemical properties of LNPs<br>• Is it optimal to minimize the PEGylated lipid content (danger of hypersensitivity reactions or anaphylactic shock)<br>• Increasing TFR significantly reduced the hydrodynamic diameter of particles<br>• Greater turbulence produced by a higher TFR may prevent neutral lipids from aggregating before encapsulating the RNA molecules<br>• Temperature had a significant role in affecting particle size LNPs were smaller as temperature increased during formulation<br>• Aqueous-phase pH affects particle size, increasingly acidic (lower) pH results in smaller particles<br>• Size was negatively correlated with a higher N/P ratio<br>• Significant parameters for size did not overlap with those for PDI<br>• PDI was affected by phospholipid type and content, ionizable lipid type, and RNA type<br>• PDI was highest in DSPC-containing LNPs formulated with low phospholipid content<br>• EE was significantly affected by temperature, phospholipid content, and ionizable content<br>• EE was generally highest at a midrange ambient temperature (20°C) with midrange phospholipid content (15%) and midrange ionizable lipid content (40%)<br>• Encapsulated RNA and PDI were significantly different between the mRNA and saRNA<br>• Both RNA types were affected by the ionizable lipid type and content, and temperature<br>• The same LNP technology can be used interchangeably between saRNA and mRNA<br>• saRNA can replace current mRNA-optimized LNP systems with relative ease and minimal optimization required in terms of process conditions<br>• Ionizable lipid type played a critical role in protein expression<br>• MC3-containing LNPs did not enable potent protein expression relative to the other ionizable lipids<br>• The highest levels were observed in LNPs containing ALC-0315, followed by those containing SM-102

	• Protein expression increased with higher ionizable lipid content and pH, and this effect was strongest when SM-102 was used<br>• Optimal Conditions for saRNA−LNP Formulation with Regards to Cellular Activation or LNP CQAs:<br>- phospholipid content (mol%): 15.9 (minimize cellular activation); 17.5 (maximize cellular activation); 17.5 (optimize CQAs)<br>- aqueous-phase pH: 4.53 (minimize cellular activation); 6 (maximize cellular activation); 5.25 (optimize CQAs)<br>- ionizable lipid type: ALC-0315 (minimize cellular activation, optimize CQAs) SM-102 (maximize cellular activation)<br>- ionizable lipid content (mol %): 45 (minimize cellular, maximize cellular activation); 35 (optimize CQAs)<br>• fixed parameters: N/P ratio: 10; phospholipid type: DOPE; DMG-PEG-2000 content: 1.25 %mol; total flow rate: 16 mL/min, ambient temperature during formulation: 20°C
[28]	• Ionisable cationic lipid (ICL):<br>- strong correlation between pKa and in vivo activity<br>- ideally pKa in the range of 6.2–6.5, neutral at neutral pH (7.4) and positively charged at acidic pH (<6.0)<br>- ICLs outside the range 6.2–6.5 display a rapid decline in hepatic gene silencing activity, emphasizing the importance of the ionization behaviour<br>• Linker<br>- linker oxygen plays a key part in transfection efficacy, affects the pKa, controls the chemical and enzymatic stability of the lipid<br>- depending on the structure, linkers are grouped into ethers, esters, amides, carbamates and disulfide groups<br>- the spacing between the headgroup and the linker (steric hindrance) impacts transfection efficiency<br>• Lipid tail:<br>- lipid tail double bond content (saturation/unsaturation), length, level of substitution and the tail structure (branched vs. linear) can affect the transfection efficiency<br>- Helper lipids<br>- the chemistry and concentration of helper lipids affect nanoparticle surface charge and impact the distribution and effectiveness of nanoparticles in specific tissue<br>- e.g. DOPC-neutral helper lipids preserved liver targeting,<br>- anionic helper lipids (phosphatidylserine, phosphatidylglycerol and phosphatidic acid) increased the distribution to the spleen<br>- DOTAP-cationic lipids shifted the protein expression to the lung<br>• Cholesterol<br>- improves intracellular delivery by promoting the fusion of the LNP with the membrane of endosomes in the presence of unsaturated lipids depending on its percentage in the composition<br>- high concentrations of cholesterol enhance the activity of cationic lipids which supports the aforementioned fusogenicity<br>• PEG-lipid<br>- PEGylation decreases particle size prevents LNP aggregation during storage and extends the shelf-life<br>- PEGylation chemistry and the density of PEG on the LNP surface can modify the pharmacokinetics and pharmacodynamics of the LNPs<br>- PEG with molecular weight ~ 2 kDa is used for LNP modification in a ratio <5%<br>• Particle size:<br>- preferential: 20–200 nm<br>- optimal for cellular uptake - about 100 nm<br>• Zeta potential (charge of the formulated LNP):<br>- crucial for sufficient exposure in the circulation and uptake into tissue<br>- high surface charge, either positive (+30 mV) or negative (-30 mV), usually results in faster blood clearance<br>- neutrally charged LNPs (from -10 to +10 mV) promote prolonged blood circulation<br>- positively charged NPs exhibited higher internalization than neutral or negatively charged NPs owing to the interaction with the cell membrane which is negatively charged, resulting in increased toxicity<br>- LNPs with near-neutral zeta potential have been predominantly used in the clinic<br>• LNP composition:<br>- ionisable lipid: 40–50 mol%, phospholipid: 10–12 mol%, cholesterol: 38–45 mol%, PEG lipid: 1–2 mol%<br>- ICL content influences the morphology and encapsulation efficiency<br>- 30% rise in the ICL molar ratio (reduction of cholesterol content) resulted in a reduction of encapsulation efficiency, although the same solid core structure was maintained<br>- cholesterol influences the morphology and the encapsulation<br>- using an inadequate molar ratio leads to a decreased encapsulation<br>• PEG lipid:<br>- higher content, decreased encapsulation efficiency<br>- smaller size as the PEG-lipid content is increased

	- LNPs with low PEG-lipid content (0.5 mol%), have the best gene silencing activity, owing to the superior endosomal release<br>• Formulation and manufacturing<br>- microfluidic technologies<br>- T-junction mixing<br>- hydrodynamic flow focusing<br>- staggered herringbone and toroidal mixer<br>• Stability profile<br>- to improve LNP-RNA stability the following strategies were used: the addition of buffers, surfactants and other excipients, effective process controls, freezing or lyophilization using appropriate cryoprotectants (sucrose, trehalose or mannitol)<br>• Delivery to the liver and beyond the liver, Biodistribution and pharmacokinetics:<br>- intravenous administration<br>- subcutaneous, intradermal and intramuscular administration<br>• oral administration
[29]	• LNP size is the most critical material attribute of stability, far ahead of the PEG proportion<br>• stability is increasing with PEG proportion) but decreasing with the LNP size<br>• PEG concentration is the most critical factor impacting transfection efficiency, and LNP size is the least influential factor<br>• DOTAP and Lecithin proportions do not appear as active factors
